# Response of Global Forest Management to Changes in Wood Demand

**DOI:** 10.1111/gcb.70573

**Published:** 2025-11-04

**Authors:** Bartlomiej Arendarczyk, Sam Rabin, Daniel Bampoh, Almut Arneth, Mark Rounsevell, Peter Alexander

**Affiliations:** ^1^ School of GeoSciences University of Edinburgh Edinburgh UK; ^2^ Climate & Global Dynamics Laboratory National Center for Atmospheric Research Boulder Colorado USA; ^3^ Institute of Meteorology and Climate Research, Atmospheric Environmental Research (IMK‐IFU) Karlsruhe Institute of Technology Garmisch‐Partenkirchen Germany; ^4^ Institute of Geography & Geo‐Ecology Karlsruhe Institute of Technology Karlsruhe Germany; ^5^ Global Academy of Agriculture and Food Systems University of Edinburgh Edinburgh UK

**Keywords:** climate change, forest management, land use modelling, socioeconomic scenarios, wood demand

## Abstract

Global wood harvests have steadily increased over the last several decades and are projected to continue growing to match demand for wood products. How forest managers respond to changes in wood demand depends not just on timber prices and production costs but also on competition with other land uses, changes in forest productivity, and land use policies. Wood demand projections are sensitive to assumptions about socioeconomic development, including population growth, economic growth, and policy changes. Using a spatially detailed, process‐based land use model (LandSyMM), we simulate global wood demand, harvests, and forest management intensity under a range of future socioeconomic (Shared Socioeconomic Pathways; SSPs) and climate (Representative Concentration Pathways; RCPs) scenarios. Wood demand is projected for each country using a price‐elastic demand system that models changes in demand for industrial roundwood and wood fuel in response to changes in countries' incomes and endogenously modelled wood prices. Competition for land between forestry and agriculture, including for food and animal feed, is explicitly represented. We find that future wood harvests and forest management intensity vary considerably between scenarios. Different regions show heterogeneous responses to changes in wood demand, with global demand increasing between 27% (SSP1‐RCP2.6) and 102% (SSP3‐RCP7.0) by 2100. The results suggest that additional wood harvests will primarily be met through intensification of forest management and an increase in potential yields arising from climate change and CO_2_ fertilisation. However, interactions between extreme events, nutrient limitations, and CO_2_‐driven productivity gains remain uncertain and are not fully captured in the modelled results. Understanding how global forest management will change and its impact on forest structure, species composition, and carbon storage is critical in addressing climate change mitigation and biodiversity protection.

## Graphical abstract

This study uses a global land use modelling framework (LandSyMM) to simulate global wood demand, harvests, and forest management intensity under a range of socioeconomic and climate scenarios. Findings indicate that global wood demand could increase between 27% and 102% by 2100. This rise in demand is expected to be met primarily through more intensive forest management and increased yields driven by climate change and CO_2_ fertilization.

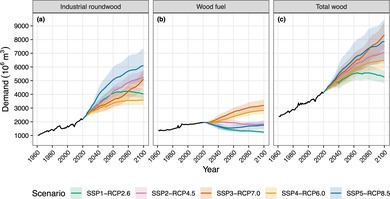

## 1 Introduction

Forests have played an important role in the economic development of societies throughout history, providing resources such as wood for construction, cooking fuel, food, and medicinal plants (Brockerhoff et al. 2017; Ritter and Dauksta 2013; Taye et al. 2021). The forestry sector contributes more than 1.5 trillion USD to global economies, and forestry products are critical inputs in many industries (Li et al. 2022). Additionally, one‐third of households globally rely on wood as their primary cooking fuel, reaching over 50% in several countries (FAO 2016). The diversity of forestry products, from paper to construction materials to wood fuel, highlights the high level of material benefits that society obtains from forests. Forest ecosystem services, and more recently, nature's contribution to people, such as carbon sequestration, soil protection, and water retention, are increasingly being put into focus as degradation and destruction of natural habitats continue at alarming rates (Brockerhoff et al. 2017; Díaz et al. 2019; FAO 2024b; Felipe‐Lucia et al. 2018; IPBES 2019; Isaac et al. 2024). While covering 31% of the world's land area, forests are the primary habitat for the majority of terrestrial species and include some of the world's most biodiverse regions (FAO 2024b; Hilton‐Taylor et al. 2009). Forest management has evolved considerably, reflecting changes in societal values, technological advancements, and scientific understanding (Ritter and Dauksta 2013). Recent discussion has focused on multi‐use management, emphasising the preservation of biodiversity, improving resilience to environmental disturbances such as climate change, and respecting the land rights of Indigenous communities (Ellison et al. 2017; Felipe‐Lucia et al. 2018; MacDicken et al. 2015).

Much of the previous work on modelling global forest management has focused on forest sector models, with prominent examples including the Global Timber Model (GTM) and the Global Forest Products Model (GFPM) (Buongiorno 2015; Favero et al. 2021; Kallio et al. 2004; Kindermann et al. 2006; Sohngen et al. 2001). Both models employ a value‐maximising approach to determine the optimal forest management decisions. The GTM maximises the net present value of the net global timber market surplus, i.e., the sum of consumer and producer surplus (Sohngen et al. 2001). Similarly, the GFPM uses a partial equilibrium approach to model the supply and demand for different forest products by maximising consumer and producer surpluses net of transaction costs (Daigneault et al. 2022). Although the GTM and GFPM include detailed representations of forest management through a combination of different forest types and forestry products, they are limited in spatial detail. GTM uses only 16 economic regions, while the GFPM comprises 180 countries with no spatial representation at the sub‐national level (Daigneault et al. 2022).

More recently, forest management has been integrated into larger modelling frameworks such as global land use models and Integrated Assessment Models (IAMs) (Doelman et al. 2018; Lauri et al. 2019; Mishra et al. 2021). Land use models consider an all‐encompassing representation of the land system, including agriculture, managed forests, natural ecosystems, and urban areas. The importance of joint modelling of forestry and other land uses stems primarily from the need to represent the trade‐offs between different land uses driven by changing demands for agricultural and forestry products. Land use models typically offer a higher spatial resolution than forest sector models and differ widely in their representation of forest management and products. For example, GLOBIOM includes six forest types and 35 forest products, while MAgPIE includes only two forest types and two forest products (Daigneault et al. 2022; Lauri et al. 2019; Mishra et al. 2021). Differences in model architecture can be a significant source of variation in results from land use models, even after accounting for initial conditions and scenario assumptions (Alexander et al. 2017; Prestele et al. 2016). Improved understanding of the inherent uncertainty in land use projections, therefore, requires comparison across a wider range of models (Daigneault et al. 2022).

One of the main uncertainties in modelling future changes in forest management stems from projections of future wood demand (Buongiorno 2015; Daigneault et al. 2022; Lauri et al. 2019; Morland et al. 2018; Nepal et al. 2021). Between 1961 and 2022, the global annual roundwood harvest increased by 58%, from 2517 million m^3^ to 3983 million m^3^ (FAO 2024a). Future projections of global wood demand vary widely depending on methodology and assumptions about population growth, economic growth, and policies relating to energy use and climate change mitigation (Daigneault et al. 2022; Lauri et al. 2019; Mishra et al. 2021; Nepal et al. 2021). The demand and supply of wood products are responsive to changes in prices and economic growth, which offers the primary way of modelling future wood demand (Morland et al. 2018). Additionally, policies aimed at increasing the share of energy production from woody biomass and shifting towards “timber cities” could increase wood demand beyond historical trends (Mishra et al. 2022; J. Zhao et al. 2022). Given the potential harm of intensive wood harvest on forest ecosystems and doubts about the carbon neutrality of energy from wood, there is much controversy regarding the net benefits of large‐scale bioenergy from forest products (Peng et al. 2023; Sterman et al. 2018). The lack of agreement about the likely trajectory of future wood demand means that novel projections of future demand are needed to help establish a better understanding of the potential evolution of global forest management. Although several past studies have looked at future forestry scenarios, the diversity of published scenarios is limited and often lacks spatial or temporal detail.

Here, we address the gaps in understanding future global forest management by exploring novel, spatially detailed projections of wood demand, forest management intensity, and wood harvests for a range of socioeconomic and climate scenarios. Using the Land System Modular Model (LandSyMM), we simulate how different countries will respond to changes in wood demand, considering factors such as changes in forest productivity from climate change and atmospheric CO_2_ concentration, competition with agriculture, and international trade. Climate change has already had a significant impact on forest productivity, with an overall increase in productivity globally and a variable pattern locally depending on factors such as water availability (Boisvenue and Running 2006). Incorporating climate and CO_2_ effects into forest sector models is therefore essential to produce realistic projections of global wood supply (Daigneault et al. 2022; Favero et al. 2021; Lauri et al. 2019). In addition, the spatial and temporal patterns of global forest management are shaped by socioeconomic factors including international trade (Zhang et al. 2020) and competition for land with agriculture (Bousfield et al. 2024). LandSyMM explicitly represents these factors, allowing us to include scenario‐specific assumptions about climate, global trade, and competition for land.

We model a distinct range of scenarios using the Shared Socioeconomic Pathways (SSPs) and Representative Concentration Pathways (RCPs) to capture the diversity of potential outcomes. We aim to contribute to the ongoing discussion on the future of forestry and its implications for climate change mitigation and biodiversity protection. By using the widely applied SSP and RCP scenario framework, we aim to make our results comparable to previous and forthcoming studies. The primary aim of this study is to assess how changes in population, GDP, and climate change could affect global wood demand and forest management over this century.

## 2 Methods

**FIGURE 1:**
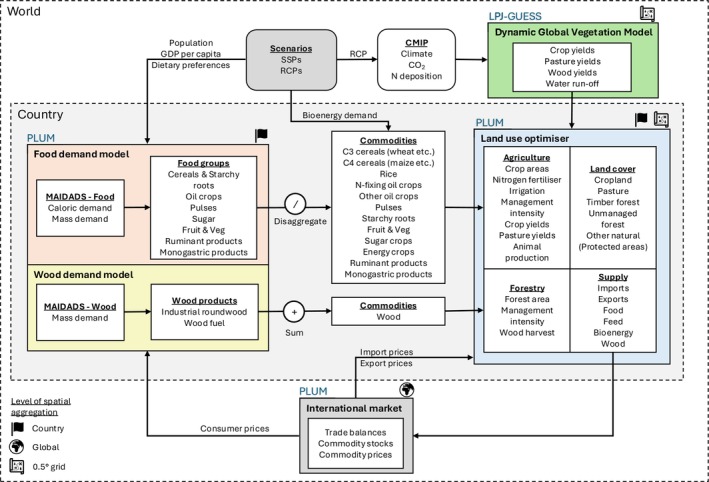
Diagram of LandSyMM architecture. The primary interactions between input data and sub‐models are shown, including: MAIDADS—a price‐elastic demand model used here to project demand for food and wood commodities (Cranfield et al. 2005; Gouel and Guimbard 2019), PLUM—a global land use model (Alexander et al. 2018), and LPJ‐GUESS—a dynamic global vegetation model (Ma et al. 2022; Olin et al. 2015; Smith et al. 2001, 2014). Input data sources include the SSP database (Riahi et al. 2017), FAOSTAT (FAO 2024a), and the CMIP project (Eyring et al. 2016).

We develop and apply a novel implementation of forest management in LandSyMM (https://landsymm.earth), a spatially detailed, process‐based global land use modelling framework. Managed forest area, forest management intensity, and wood harvesting are simulated globally on a 0.5‐degree grid with an annual time step using least‐cost optimisation. Wood demand is projected country by country using a price‐elastic demand system fitted to historical wood consumption and prices. Changes in wood demand are driven by changes in population and GDP per capita for a range of Shared Socioeconomic Pathways (SSPs), as well as changes in wood prices calculated endogenously. LandSyMM couples a dynamic global vegetation model (LPJ‐GUESS) with a global land use model (PLUM) to simulate country‐level demand, international commodity trade, spatially explicit yields, food production, energy crops, and wood harvests (Figure 1).

### 2.1 Land Use Modelling Framework

LPJ‐GUESS is a process‐based dynamic global vegetation model that simulates ecosystem processes, including vegetation and soil carbon dynamics, the nitrogen cycle, and plant physiological responses to climate change, atmospheric CO_2_, and disturbance (Ma et al. 2022; Olin et al. 2015; Smith et al. 2001, 2014). We used LPJ‐GUESS version 4.1 to generate potential yield responses for 10 crop functional types (CFTs) and pasture under different climate, nitrogen fertilisation, and irrigation regimes (Alexander et al. 2018; Ma et al. 2022). A second‐generation bioenergy crop (*Miscanthus*) was modelled as a C4 cereal CFT. Details of forest yield simulations can be found in section “Wood yields”. Simulations were driven by bias‐corrected climate data from the Inter‐Sectoral Impact Model Intercomparison Project (ISIMIP) for the MRI‐ESM2‐0 general circulation model (Eyring et al. 2016; Yukimoto et al. 2019). Simulated crop yields were calibrated to observed country average yields for the period 2005–2014, accounting for nitrogen fertiliser use, irrigation, and GDP per capita as a proxy for other inputs such as pesticide use and mechanisation (Figure S1).

The Parsimonious Land Use Model (PLUM) incorporates crop yields, wood yields, and water run‐off for irrigation from LPJ‐GUESS to simulate land use and land use change from changes in demand for food commodities, bioenergy (Alexander et al. 2018), and wood (described in this study). Food demand is projected using a Modified An Implicit Directly Additive Demand System (MAIDADS) (Cranfield et al. 2005; Gouel and Guimbard 2019), which uses per capita income (exogenously specified) and commodity prices (modelled endogenously in PLUM) to calculate demand for seven food groups. A separately fitted MAIDADS is also used to project wood demand, as described later. Regional demand for second‐generation bioenergy was taken from the IIASA SSP Database (Riahi et al. 2017) and disaggregated to country level based on potential *Miscanthus* production in the baseline year (2020) using yields from LPJ‐GUESS. The initial land cover distribution was taken from HILDA+ (Winkler et al. 2021) and mapped to the PLUM land cover classes.

During each annual time step, PLUM uses least‐cost optimisation for each country to determine land use factors including cropland, pasture, and forest area, fertilizer input, irrigation, a crop management intensity factor (representing, for example, pesticide use, phosphorus fertilizer, and mechanisation), and forest management intensity. Crop yields are interpolated for a continuous range of fertilizer application rates and irrigation using yield tables generated by factorial experiments in LPJ‐GUESS. Irrigation is constrained at the water basin level by the estimated surface water runoff modelled by LPJ‐GUESS. Where sufficient food, bioenergy, and wood to meet demand are not met from domestic production, the balance is imported. A single international market allows countries in PLUM to import and export commodities, with prices adjusted annually based on the net balance of imports and exports.

### 2.2 Scenarios

We assessed results for five scenarios, comprising Tier 1 combinations of SSPs and RCPs: SSP1‐RCP2.6, SSP2‐RCP4.5, SSP3‐RCP7.0, SSP4‐RCP6.0, and SSP5‐RCP8.5 (Table 1). We used population and GDP (2017 PPP) projections from Koch and Leimbach (2023) who harmonise and update projections from the IIASA SSP database (Riahi et al. 2017) with recent demographic and economic changes. Changes in global dietary preferences were modelled as a shift from historical dietary patterns towards a healthier and more sustainable diet based on the EAT Lancet recommendation (Willett et al. 2019) (Table S3). The degree of shift in dietary preferences was determined by the authors' interpretation of the scenario narratives with a 100% shift in SSP1‐RCP2.6, a 50% shift in SSP4‐RCP6.0, and no change in preferences in SSP2‐RCP4.5, SSP3‐RCP7.0, and SSP5‐RCP8.5. To simulate uncertainty in modelled outcomes, simulations were repeated with stochastically sampled input parameters from distributions consistent with scenario narratives from O'Neill et al. (2017). The sampled parameter distributions are visualised in Figure S2.

**TABLE 1:** Scenario narratives summarised from O'Neill et al. (2017).

Scenario narratives	
Scenario	Summary of scenario narrative
SSP1‐RCP2.6	A green‐growth scenario with high economic growth, low population growth, and a high‐level of international cooperation and technological development
SSP2‐RCP4.5	A business‐as‐usual scenario in which historical trends in socioeconomic development are continued
SSP3‐RCP7.0	A scenario characterised by regional rivalry, with high population growth, low economic growth and lack of international cooperation
SSP4‐RCP6.0	A scenario with high international inequality, with diverging trends in population and economic growth between developed and developing countries
SSP5‐RCP8.5	A fossil‐fuelled economic growth scenario with minimal effort to mitigate climate change

### 2.3 Wood Yields

**FIGURE 2:**
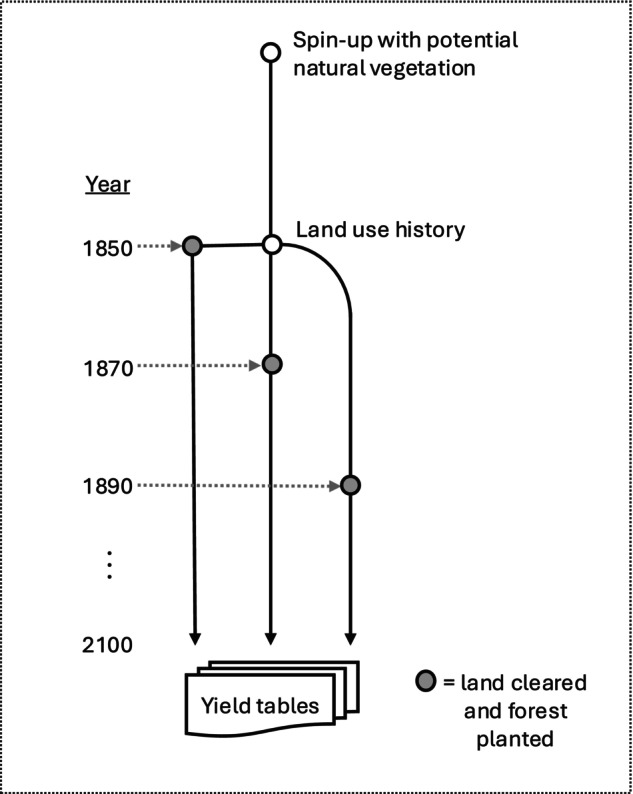
Diagram showing simulation of potential wood yields in LPJ‐GUESS. Following spin‐up with potential natural vegetation and subsequently historical land use (white circles), all land cover was converted to pasture for 1 year and then forest stands were established at 20‐year intervals (grey circles). Yield tables were generated each year until 2100, containing potential wood harvest from clear‐cutting of the forest stands.

We conducted LPJ‐GUESS simulations to generate yield tables of expected wood yields. Previously within LandSyMM, LPJ‐GUESS was only used to simulate agricultural crop yields, irrigation water requirements, and to constrain water availability (Alexander et al. 2018; Rabin et al. 2020). For each grid cell, the wood yield tables provide information on how much wood can be expected from a clear‐cutting of a forest of a certain age. Yield tables were created every 20 years, starting from 1850 up to 2090, with each continuing through to 2100 (Figure 2). This was done to account for the effects of changing climate and atmospheric CO_2_ concentration.

Following a spin‐up with no human land use and only potential natural vegetation, we began a run in 1850 with land use from the Land‐Use Harmonization 2 (LUH2) project (Hurtt et al. 2020). Forest management (e.g., logging and thinning) was not represented in this LPJ‐GUESS run and, therefore, all forests were treated as unmanaged. This run continued through the historical period and into the future, with land‐use fractions derived from LUH2 data for the different SSP‐RCP scenarios. Historical and projected land use from LUH2 was included to account for the impact of land use and land use change on forest growth (e.g., through soil carbon impacts). Every 20 years, a new run was initialized (branched off), with its initial state taken directly from the main run (see branching points represented by grey circles in Figure 2). Each branch consisted of one full year of 100% pasture cover, followed by the establishment of 100% forest cover, which remained constant for the remainder of the simulation.

Each year, LPJ‐GUESS calculated the wood yield from a hypothetical clear‐cutting harvest. The yield was calculated as the sum of biomass consisting of 100% trunk (65% of the wood pool), 95% of twigs and branches (13% of the wood pool), and 10% coarse roots (22% of the wood pool). Yields were converted from biomass to wood volume using a conversion factor of 0.3 tC m^−3^ (Hurtt et al. 2020). For these runs, we allowed disturbance, including fire (SIMFIRE‐BLAZE model), and all possible Plant Functional Types (PFTs) were allowed to grow within the model (Rabin et al. 2020; Smith et al. 2014). Yields were based on native potential natural vegetation. Hence, the effect of different tree species compositions on yields was not modelled here (e.g., monoculture vs. multi‐species plantations).

### 2.4 Wood Demand Model

For each country, wood demand was calculated using MAIDADS (Cranfield et al. 2005), following a similar procedure to Gouel and Guimbard (2019) used here for modelling food demand. The model was fitted for 2017 using apparent wood consumption and prices from FAOSTAT (FAO 2024a), and GDP per capita (2017 $ PPP) from the International Comparison Project (The World Bank 2020). Apparent consumption—equal to production plus imports minus exports—was used as a proxy of demand (Equation 1). The FAOSTAT forestry products database reports the production and trade of raw, intermediate and final wood products. Items reported in tonnes (wood pulp, paper and paperboard, wood pellets and other agglomerates, and wood charcoal) were converted to cubic meters using conversion ratios from UNECE, Eurostat, and FAO (2022) (Table 2). We assumed that all by‐products from wood processing are accounted for in the reported production, for example, in wood chips, particles and residues. Finally, the industrial roundwood or wood fuel equivalent volume of each item was calculated by simply assuming a one‐to‐one volume equivalence. While we did not adjust production values for potential losses, we found that this approach gave a good approximation to the total raw material input—2033 million m^3^ of industrial roundwood estimated from processed products compared to the actual 1952 million m^3^ harvested in 2017.

**TABLE 2:** Mapping of FAOSTAT wood items to PLUM commodities. Items reported in tonnes were converted to cubic meters using conversion ratios from UNECE, Eurostat and FAO (2022).

Wood product mapping		
FAOSTAT item	PLUM commodity	Conversion ratio (m^3^ t^−1^)
Industrial roundwood	Industrial roundwood	—
Sawnwood	Industrial roundwood	—
Wood‐based panels	Industrial roundwood	—
Veneer sheets	Industrial roundwood	—
Wood chips, particles and residues	Industrial roundwood	—
Wood pulp	Industrial roundwood	1.48^a^
Paper and paperboard	Industrial roundwood	1.48^a^
Wood fuel	Wood fuel	—
Wood pellets and other agglomerates	Wood fuel	1.38^b^
Wood charcoal	Wood fuel	5.99

^a^ From pulpwood.

^b^ From wood fuel.

We used a mass balance approach to calculate apparent wood consumption in each country for industrial roundwood and wood fuel (Equation 1). Here, we assumed that apparent consumption equals the sum of the within‐country harvest of raw material (industrial roundwood or wood fuel) and net trade of raw and processed wood products. Harvest, production, and trade statistics were first corrected for underreporting and inconsistencies (see Supporting Information). Then, for each country:

(1) $$\text{consumption}=\text{harvest}+\sum_{i}\text{import}s_{i}-\sum_{i}\text{export}s_{i}$$

where consumption is apparent consumption, harvest is the amount of industrial roundwood or wood fuel harvested within the country, $\text{import}s_{i}$ and $\text{export}s_{i}$ are imports and exports of item i. Table 1 shows the categorisation of reported FAOSTAT wood items into the two PLUM wood commodities (industrial roundwood and wood fuel).

For each country, we used trade values and trade quantities to estimate the prices of wood products (FAO 2024a). First, we aggregated trade values and quantities, converting item quantities into their industrial roundwood or wood fuel volume as detailed previously. We calculated import and export prices by dividing the trade values by their respective trade quantities. Subsequently, commodity prices were calculated as an average of import and export prices weighted by import and export amounts. For countries where no data was available, missing prices were replaced with median global prices.

**FIGURE 3:**
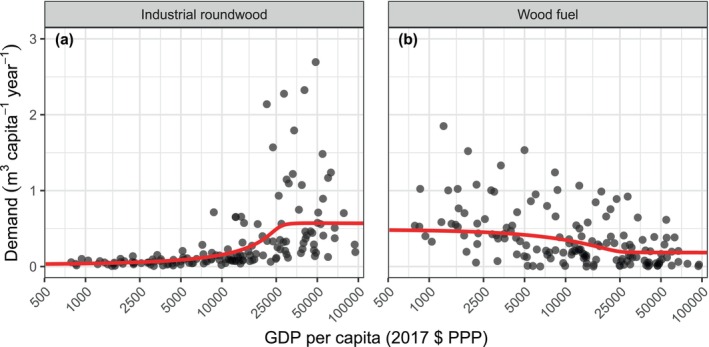
Reported (black points) and predicted (red lines) per capita wood demand for (a) industrial roundwood and (b) wood fuel. Reported demand was calculated as the sum of domestic production and net imports of wood products. Predicted demand plotted here assumes constant average 2017 prices.

During a model run, MAIDADS predicts per capita demand for industrial roundwood and wood fuel using GDP per capita, prices, and fitted parameters that control consumption preferences as a function of income (Table S1). Per capita wood demand is then multiplied by country population to give the total wood demand for the country. Wood prices are calculated endogenously based on the global trade balance, using the same method as for agricultural products (Alexander et al. 2018). The price elasticity of wood demand depends on the income level, which gives rise to complex and dynamic changes in both the aggregate wood demand and preference for roundwood or fuel wood (Figure S3). Figure 3 shows the observed wood demand and model predictions, assuming average 2017 prices.

### 2.5 Forest Management in PLUM

The initial 2020 distributions of forests managed for timber production (hereafter, timber forest) and unmanaged forests were taken from HILDA+ (Winkler et al. 2021). In HILDA+, the timber forest area was defined as forested land with any signs of harvesting, such as logging and clear‐cutting, as per Lesiv et al. (2022). In LandSyMM, timber forests broadly represent managed wood‐producing forests, ranging from intensive forest plantations to semi‐managed forests with occasional harvests. Forest management is represented by a continuous management intensity variable r (Equation 2). The management intensity can be interpreted as the harvest frequency or, equivalently, the reciprocal of the rotation period. For example, an intensity of 0.02 corresponds to a 50‐year rotation period. However, we can also interpret the intensity as the fraction of a grid cell harvested each year. Harvest is simulated through clear‐cutting and replanting of trees. The expected annualised wood harvest is calculated for each location based on the management intensity and parameters calculated using yield tables from LPJ‐GUESS. Although demand is calculated separately for industrial roundwood and wood fuel, no distinction between wood products is made in the harvesting or trade of wood. Hence, for each grid‐cell,

(2) $$y=y_{\max}{\left(1-e^{\frac{k}{r}}\right)}^{p}r$$

where y is the annualised wood harvest, r is the management intensity, and $y_{\max}$, k, and p are estimated from yield tables using the Gauss‐Newton method. The value of r is determined by least cost optimisation for each country, such that total wood harvests equal demand, accounting for any imports and exports. The three parameters, $y_{\max}$, k, and p are re‐estimated each time step using a different portion of the wood yield tables, based on a sliding‐window approach such that yields are continuously updated to reflect the changing climate and atmospheric CO_2_ levels. The intensity variable r is constrained between 0.00625 and 0.1, the former value representing the time scope of the yield tables (160 years) and the latter based on reported forest rotation periods (Del Lungo et al. 2006) and the authors' judgement. The cost of forest management is equal to c·r where c is the forest establishment and management cost per hectare. Higher management intensities are therefore associated with a higher annual forest management cost. A single global forest management cost was assumed, fixed through time but varying with scenario (see “forest management cost” parameter in Figure S2).

Wood harvesting decisions are integrated into the land use optimiser in PLUM, which also includes food, feed, and energy crop production (Figure 1). Land use decisions in PLUM are made through least‐cost optimisation (Alexander et al. 2018). At each time step, the model calculates industrial roundwood and wood fuel demand individually for each country based on GDP per capita and population from the SSP scenario and the endogenous wood price. At the land use optimisation step, the model is constrained to meet demand through harvest in each country, net of imports and exports. Wood harvesting can be altered through the expansion and contraction of timber forest areas and through changes in forest management intensity. Changes in imports and exports are constrained through absolute limits (as a percentage of the current trade level) and a small cost associated with changing net import levels. The absolute limits are set loosely to avoid over‐constraining the model while ensuring model stability. At the end of each timestep, net import levels are aggregated, and the wood commodity price is updated based on the change in the global stock level following the same method as for food and feed commodities (Alexander et al. 2018).

## 3 Results

### 3.1 Present Distribution of Wood Harvests

**FIGURE 4:**
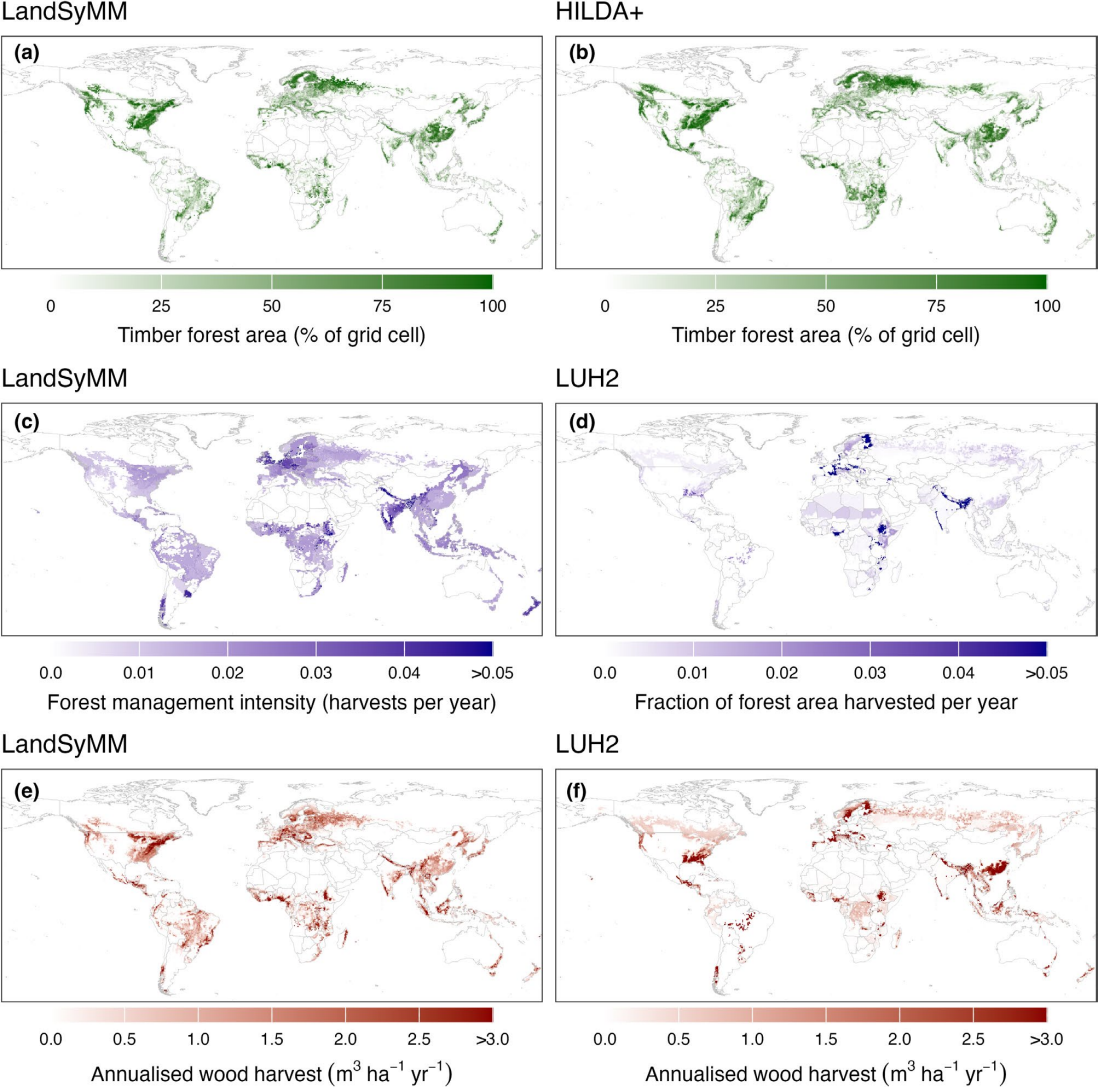
Simulated distribution of (a) timber forest area, (c) forest management intensity, and (e) wood harvests in 2020. Timber forest area is shown as a percentage of the grid cell area. Forest management intensity represents the frequency of wood harvest. Wood harvests are shown as annualised harvest per grid cell area. Reference distribution of (b) timber forest area from HILDA+ (Winkler et al. 2021), (d) average fraction of forest area harvested per year between 2005 and 2015 from LUH2 (Hurtt et al. 2020), and (f) annualised wood harvest between 2005 and 2015 from LUH2. Map lines delineate study areas and do not necessarily depict accepted national boundaries.

We estimate that 1460 Mha of forest was managed for timber production (timber forest) in 2020, representing 36% of the total global forest area (4102 Mha). For comparison, the FAO (2020) estimates that approximately 30% of global forest area is used primarily for wood production. The simulated distribution of timber forests in 2020 (Figure 4a) corresponds closely to the timber forest layer from HILDA+ which was used to initialise the model (Figure 4b). However, there are some minor differences given that LandSyMM is not constrained to reproduce the initial land cover during spin‐up. These differences primarily correspond to grid cells with very low potential wood yields, for example, in the boreal and arid regions. During spin‐up, LandSyMM abandons the initialised timber forest in these regions, leaving natural vegetation. The initial timber forest layer may also include grid cells misclassified as timber forests. These grid cells are then abandoned or converted to other land uses which the model determines to be more suitable, for example, for food production. The distribution of timber forests in LandSyMM also shows good correspondence with the distribution of managed forests reported in Schulze et al. (2019) and Lesiv et al. (2022).

We find that areas of high forest management intensity are concentrated in regions with high timber demand and limited land availability, such as Europe and South Asia (Figure 4c). Management intensity is particularly high in densely populated countries such as the United Kingdom and India, with an area‐weighted average intensity of 0.038 and 0.034, respectively, compared to the global average of 0.018. While the Americas are major producers of wood globally (FAO 2024a), forest management intensities are lower in this region due to extensive forest cover. This includes major wood producers such as the United States and Brazil, with mean area‐weighted intensities of 0.013 and 0.015, respectively. We also find areas of high forest management intensity in equatorial Africa, driven by high demand for wood fuel.

We compared simulated forest management intensity (Figure 4c) and wood harvests (Figure 4e) against the LUH2 dataset (Hurtt et al. 2020), which includes global wood harvest maps downscaled from country‐level FAO statistics (Figure 4d,f). To make the LUH2 data comparable with our results, biomass harvest was converted to wood volume using a conversion factor of 0.3 tC m^−3^ (Hurtt et al. 2020). Additionally, we show the average fraction of forest area harvested per year between 2005 and 2015 from LUH2 (Figure 4d). Some similarities can be seen between LandSyMM and LUH2 in the broad global distribution and hotspots of harvested areas and harvest volumes. For wood harvests, while the grid‐level correlation between our results and LUH2 is low (Pearson correlation coefficient = 0.36), we note that the LUH2 dataset appears to contain unusual patterns (e.g., in Europe and Brazil) which suggest data limitations. For Europe, Verkerk et al. (2015) show better grid‐level agreement (Pearson correlation coefficient = 0.64) with our data for wood harvests than LUH2. On the country level, the simulated wood harvests show good agreement with reported harvests from FAOSTAT (Pearson correlation coefficient = 0.94; Figure S4).

### 3.2 Wood Demand

**FIGURE 5:**
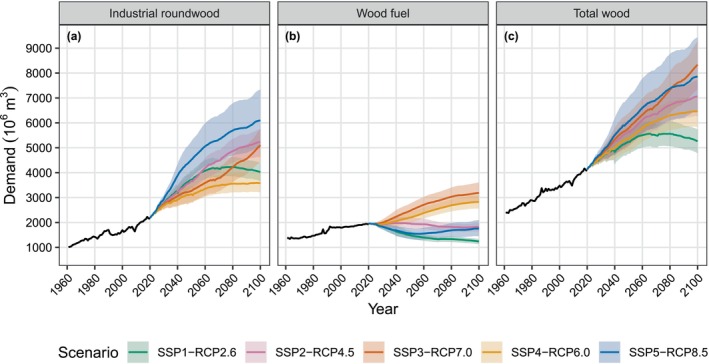
Global historical (solid black lines) and simulated median future (coloured lines) wood demand for (a) industrial roundwood, (b) wood fuel, and (c) total wood. Shaded areas represent the 90th percentile confidence intervals from a Monte Carlo simulation (*n* = 30 for each scenario). Historical demand was calculated from FAOSTAT (FAO 2024a).

The results indicate a 33%–60% increase in total global wood demand by 2060 across all scenarios (Figure 5; Table S4). The rapid growth in demand in SSP5‐RCP8.5 is driven primarily by an increase in GDP per capita, with most countries converging towards high‐income demographics by the end of the century. In contrast, the increase in wood demand in SSP3‐RCP7.0 is primarily due to rapid population growth. In SSP5‐RCP8.5, the growth in total wood demand comes solely from an increase in demand for industrial roundwood, while demand for wood fuel decreases. The divergence in demand for industrial roundwood and wood fuel is seen in scenarios where the average global income increases at or above historical rates (SSP1‐RCP2.6, SSP2‐RCP4.5, SSP5‐RCP8.5). Where income growth is weaker, in SSP3‐RCP7.0 and SSP4‐RCP6.0, continued growth in demand for both industrial roundwood and wood fuel is seen.

By 2100, the scenarios show significant differences in global wood demand, with demand increasing between 27% (SSP1‐RCP2.6) and 102% (SSP3‐RCP7.0) from 2020 (Figure 5; Table S4). The three scenarios with the lowest total wood demand, SSP1‐RCP2.6, SSP2‐RCP4.5, and SSP4‐RCP6.0, show demand stabilizing towards the end of the century. In all scenarios except SSP1‐RCP2.6, industrial roundwood demand continues to increase throughout the coming century. The trend in wood fuel demand is more variable, with a continuing increase in demand in SSP3‐RCP7.0 and SSP4‐RCP6.0, and decreasing or stable demand in SSP1‐RCP2.6, SSP2‐RCP4.5, and SSP5‐RCP8.5. In SSP1‐RCP2.6, total wood demand decreases after 2080, correlating with a shrinking global population. In SSP1‐RCP26 and SSP5‐RCP8.5, a rapid increase in country incomes in the first half of the century leads to fast growth in demand for industrial roundwood and a sharp fall in demand for wood fuel. SSP4‐RCP6.0 shows a unique pattern of very low growth in industrial roundwood demand and a high increase in wood fuel demand. This reflects the unequal socioeconomic development in this scenario, where lower‐income countries rely on wood fuel for their energy needs. In line with a business‐as‐usual narrative, demand is broadly intermediate in SSP2‐RCP4.5 compared to other scenarios.

**FIGURE 6:**
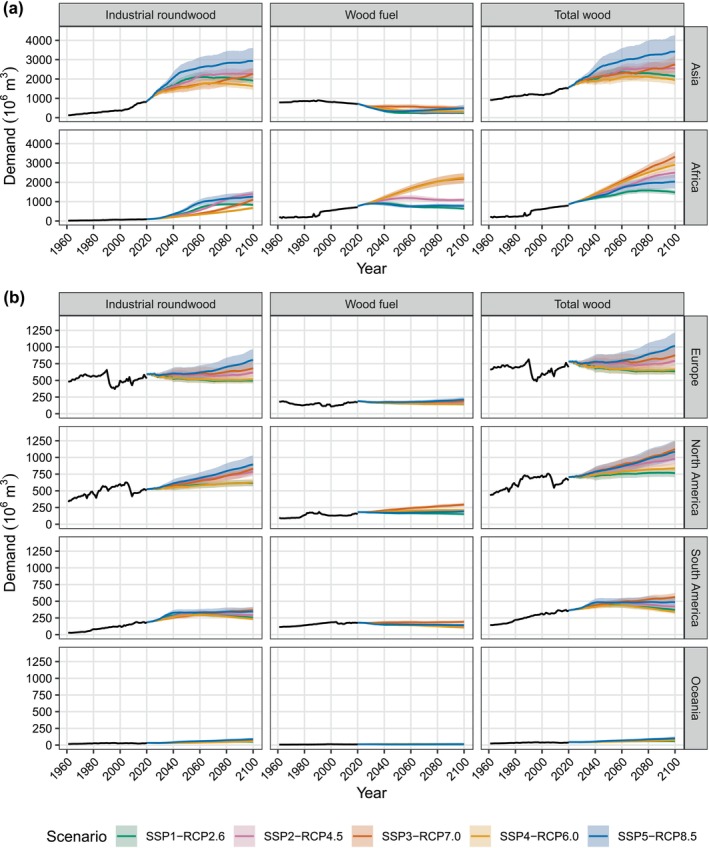
Historical (solid black lines) and simulated future (coloured lines) wood demand for industrial roundwood, wood fuel, and total wood (columns), split by region (rows). Shaded areas represent 90th percentile confidence intervals from a Monte Carlo simulation (*n* = 30). Historical demand was calculated from FAOSTAT (FAO 2024a). For visual clarity, different vertical scales are used for (a) Asia and Africa, and (b) Europe, North America, South America, and Oceania.

In all scenarios, most of the projected increase in global wood demand can be attributed to Africa and Asia (Figure 6). In SSP2‐RCP4.5, of the 2935 million m^3^ median increase in global wood demand, 987 million m^3^ comes from Asia and 1636 million m^3^ from Africa, representing 89% of the global increase. In SSP4‐RCP6.0, the increase in global wood demand can be almost entirely attributed to Africa, with demand being flat or decreasing in other regions. In Africa, North America, and South America, the highest wood demand in 2100 is seen under SSP3‐RCP7.0, whereas in Asia and Europe, this is under SSP5‐RCP8.5. The only regions to show a decline in total wood demand by 2100 are Europe in SSP1‐RCP2.6 and SSP4‐RCP6.0, and South America in SSP4‐RCP6.0. Demand for industrial roundwood is projected to increase in all regions apart from Europe in SSP1‐RCP2.6 and SSP4‐RCP6.0. The strongest increase is found in Africa and Asia, particularly under SSP5‐RCP8.5 and SSP2‐RCP45. Demand for wood fuel shows a less consistent trend across regions and scenarios. A decrease in wood fuel demand can be seen in most regions in SSP1‐RCP2.6. An increase in wood fuel demand is seen in SSP3‐RCP7.0 and SSP4‐RCP6.0, with the largest increases in Africa and North America.

### 3.3 Prices

**FIGURE 7:**
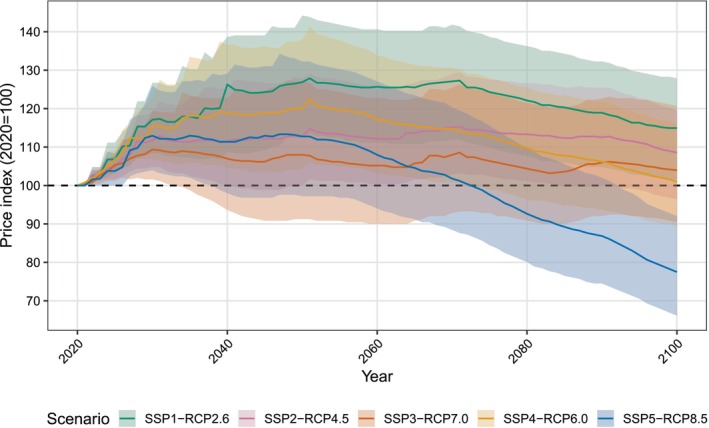
Wood price index (2020 = 100). Median prices are shown by solid lines and shaded areas represent the 90th percentile confidence intervals from a Monte Carlo simulation (*n* = 30).

The wood price index shows an initial increase in all scenarios in the first half of the century, followed by a decline in SSP1‐RCP2.6, SSP4‐RCP6.0, and SSP5‐RCP8.5 (Figure 7). In most scenarios, the price of wood peaks around 2050. Higher prices in SSP1‐RCP2.6 and SSP4‐RCP6.0 are partly responsible for the lower total demand in these scenarios, particularly in higher‐income countries for which the elasticity of demand is more negative (Figure S3). In SSP1‐RCP2.6, the high cost of forest management leads to the largest increase in the price index compared to other scenarios, with a peak price index of 128 (90% CI: 114–144) in 2051. Conversely, despite a high wood demand, SSP5‐RCP8.5 shows a substantial decrease in the price index, falling to 77 (66–92) by 2100. This can be attributed to the low cost of forest management in this scenario and increased forest yields due to CO_2_ fertilisation resulting from high carbon emissions. Changes in wood prices appear to be correlated with carbon emissions (as defined by the RCP), suggesting a positive impact of climate change and increased CO_2_ levels on the global wood supply. However, this pattern is confounded by the different socioeconomic assumptions and parameterisation of each scenario, which can also strongly impact the global wood market.

### 3.4 Changes in Global Forest Area

**FIGURE 8:**
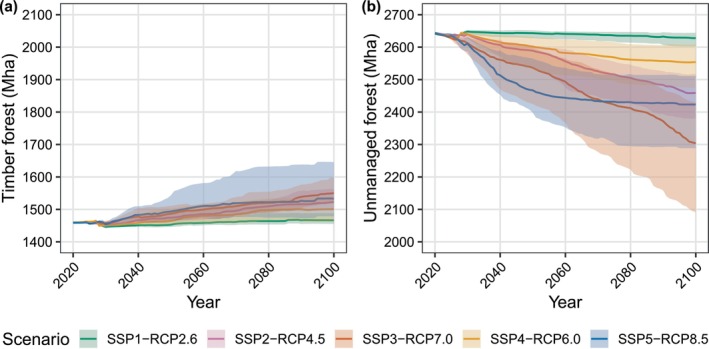
Simulated global land cover from 2020 to 2100 for (a) timber forest and (b) unmanaged forest. Median areas are shown by solid lines, and shaded areas represent the 90th percentile confidence intervals from a Monte Carlo simulation (*n* = 30).

Results suggest that growth in global wood demand will lead to a relatively modest increase in timber forest area compared to the loss of unmanaged forests due to agricultural expansion (Figure 8; Table S5). In SSP3‐RCP7.0, despite a 102% increase in wood demand by 2100, timber forest area increases by only 6.3% (90% CI: 4.1% to 9.5%). Other scenarios show a smaller change in the global timber forest area by 2100 compared to SSP3‐RCP7.0 (Table S5). While timber forest expansion primarily follows increases in global wood demand, it is also influenced by factors including land use costs, changes in forest productivity, and competition for land with agriculture. In SSP1‐RCP2.6, low wood demand and a high land conversion cost result in only minimal expansion of timber forests, while the opposite factors result in faster expansion in SSP3‐RCP7.0 and SSP5‐RCP8.5.

Most of the change in global forest area in these scenarios is driven by the loss of unmanaged forests. SSP3‐RCP7.0 shows the largest decrease in unmanaged forest area, with a median loss of −12.8% (−20.8% to −8.1%) between 2020 and 2100. This is driven by cropland and pasture expansion due to high food demand from population growth. A substantial loss of unmanaged forests is also seen in SSP5‐RCP8.5, although most of this occurs by 2060. As expected from the environmentally oriented scenario narrative in SSP1‐RCP2.6, we see minimal unmanaged forest loss. In SSP2‐RCP4.5 and SSP4‐RCP6.0, unmanaged forest loss is intermediate compared to other scenarios.

**FIGURE 9:**
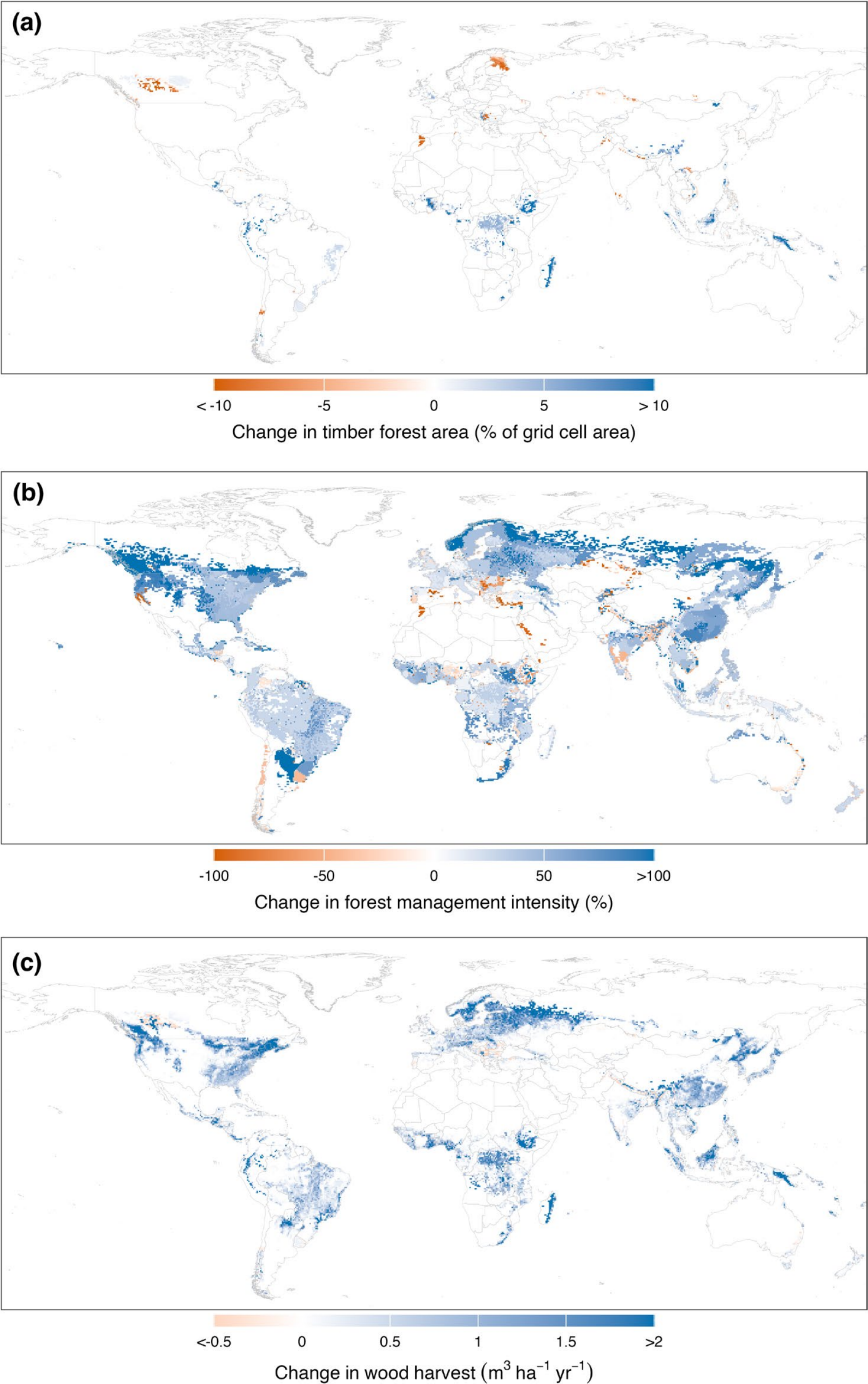
Change in (a) timber forest area, (b) forest management intensity, and (c) wood harvests between 2020 and 2100 for SSP2‐RCP4.5. Map lines delineate study areas and do not necessarily depict accepted national boundaries.

We find that nearly all expansion of timber forest area occurs in the tropics and subtropics (Figure 9a; see Figure S5 for all scenarios). Between 2020 and 2060, most scenarios show expansion in east‐central Africa, southeast Asia, and western South America. Some loss of timber forest area occurs in boreal countries such as Canada and Finland. Overall, the spatial patterns are similar in each scenario and differ primarily in the extent of change rather than location. These patterns diverge more by the end of the century. Further expansion of timber forest is seen in Central Africa in SSP2‐RCP4.5, SSP3‐RCP7.0, and SSP5‐RCP8.5, but not in SSP1‐RCP2.6 and SSP5‐RCP8.5. Expansion of timber forest also extends to other areas, including Brazil and Canada.

### 3.5 Changes in Forest Management Intensity and Harvests

In contrast to previous studies, we report spatially explicit changes in forest management intensity under each SSP‐RCP scenario (Figure 9b; see Figure S6 for all scenarios). Between 2020 and 2100, the global area‐weighted forest management intensity increases between 8% (SSP1‐RCP2.6) and 67% (SSP3‐RCP7.0) (Table S6). The degree of increase in intensity generally correlates with changes in global wood demand. Where demand growth is highest, such as in SSP3‐RCP7.0 and SSP5‐RCP8.5, we observe a large increase in forest management intensity, with many areas showing an increase of more than 100%. In SSP1‐RCP2.6, where growth in demand is the weakest, much of the world shows a drop in forest management intensity by the end of the century.

We find that changes in intensity are not uniform, with different areas experiencing both increases and decreases in intensity across time. However, some regions show a more consistent response across scenarios. For example, North America, northern Europe, Asia, and south‐east China consistently show increased forest management intensity. A decrease in intensity is seen in arid areas such as the Mediterranean basin, relating to a decline in forest productivity. These complex patterns arise from multiple factors modelled explicitly in LandSyMM, including changes in wood yields, demand, land competition from agriculture, and international trade.

Irrespective of the scenario, we find a consistent increase in wood harvests in nearly all timber forest areas (Figure 9c; see Figure S7 for all scenarios). Between 2020 and 2100, global wood harvests increase between 29% (SSP1‐RCP2.6) and 103% (SSP3‐RCP7.0) (Table S5). Strong increases in wood harvests are seen in the boreal regions, driven by increases in forest productivity, as well as in parts of the tropics due to local demand growth.

### 3.6 Factor Contributions to Changes in Wood Harvests

**FIGURE 10:**
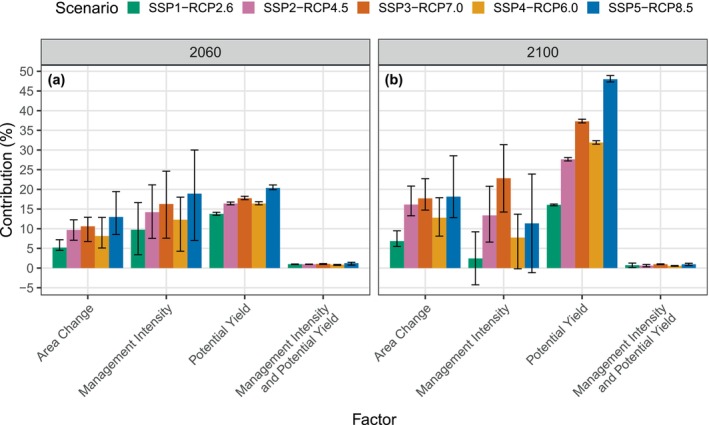
Factor decomposition of global changes in wood harvests between (a) 2020 and 2060 and between (b) 2020 and 2100. Each bar represents the percentage of the change in global wood harvests that can be attributed to one of each factor: Timber forest area change, management intensity change, and potential yield change. “Management Intensity and Potential Yield” represents the non‐linear interaction between changes in management intensity and potential yields as defined by Equation (2). Contributions are multiplicative. Bars show the median contribution and error bars show the 90% confidence interval across 30 ensemble members for each scenario.

We decomposed the contribution of different factors to changes in global wood harvest between two periods: 2020–2060 and 2020–2100 (Figure 10). Here, we use “potential yield” to refer to the effect of changes in the yield function (Equation 2) fitted from yield tables generated by LPJ‐GUESS. Increases in potential wood yields driven by climate change and atmospheric CO_2_ concentration are the largest contributor to increases in wood harvests in all scenarios. Changes in potential yields are driven by and correlated with the RCPs, with the largest increase shown in SSP5‐RCP8.5 (48.0%; 90% CI: 47.3% to 88.9%) and the smallest in SSP1‐RCP2.6 (16.1%; 16.8% to 16.3%). In all scenarios, management intensity is a stronger contributor than area change to increasing wood harvests between 2020 and 2060, but this becomes more variable between 2020 and 2100.

## 4 Discussion

The increase in future wood demand across the range of scenarios modelled here is generally consistent with results from similar studies (Daigneault et al. 2022; Lauri et al. 2019; Mishra et al. 2021; Nepal et al. 2021). Using GLOBIOM, Lauri et al. (2019) found comparable trends in global demand for industrial roundwood and wood fuel for the five SSPs. The authors found that demand for industrial roundwood increases in all scenarios, while demand for wood fuel increases in SSP3‐RCP7.0 and SSP4‐RCP6.0 but decreases in SSP1‐RCP2.6, SSP2‐RCP4.5, and SSP5‐RCP8.5 (Lauri et al. 2019). Based on results from three models (GTM, GFPM, and GLOBIOM), Daigneault et al. (2022) also found a consistent increase in global wood harvest across a wide range of SSP‐RCP combinations, with the highest wood harvest in SSP5‐RCP8.5. For other SSPs, the relative trends in wood harvest were strongly related to the RCP, for example, with SSP1‐RCP2.6 showing the second highest harvest in the baseline no‐mitigation scenario and the lowest in RCP1.9 (Daigneault et al. 2022). Mishra et al. (2021) only included SSP2‐RCP4.5 in their analysis but found the same pattern of increasing global demand for industrial roundwood and decreasing demand for wood fuel throughout this century.

Previous studies focus on regional aggregate wood demand and harvests but do not report changes in forest management intensity (Daigneault et al. 2022; Lauri et al. 2019; Mishra et al. 2021). In contrast, we produce gridded 0.5‐degree maps of forest management intensity under each scenario. On a regional level, Luo et al. (2024) found that an increase in forest management intensity will be the primary driver of increases in future wood harvests. This finding mirrors our results, which show that despite a large increase in wood demand, timber forest area remains relatively stable, and forest productivity is increased primarily through higher management intensity and potential yields due to climate change and higher atmospheric CO_2_ concentration. However, previous studies suggest that both increases and decreases in forest areas are possible under growing wood demand (Daigneault et al. 2022; Mishra et al. 2021). For Latin America, Favero et al. (2022) found that while plantation forest areas increase in each SSP in line with growth in timber production, the total forest area declines due to loss of unmanaged forests to agriculture. We observe a similar pattern globally, where the expansion of timber forests is relatively minor compared to the loss of unmanaged forests due to agricultural expansion. Differences in findings can be attributed to different model assumptions, such as the cost of land cover conversion, cost of management intensification, and land use policies (such as those encouraging reforestation or restricting deforestation). More broadly within land use models, model architecture can also significantly impact land use outcomes even when accounting for initial conditions and scenario assumptions (Alexander et al. 2017).

While changes in forest management intensity and area are directly influenced by domestic demand, international trade can also play an important role in determining the global pattern of wood harvests. For many countries, the global wood harvest footprint is considerably greater than the domestic harvest footprint, and 42% of harvested forest area is used to satisfy foreign demand for wood products (Arto et al. 2022; Zhang et al. 2020). Our findings show that despite a fall in regional demand under some scenarios, wood harvests continue to increase in these regions due to increased exports to the global market. The presence of telecouplings in the forest sector means that sustainable forest management policies such as low‐impact logging could end up displacing wood harvests to other regions (Searchinger et al. 2022).

Changes in wood demand will have important implications for global forest management practices. If global wood demand continues to grow at a high rate, such as in SSP3‐RCP7.0 and SSP5‐RCP8.5, the intensification of wood harvests will likely be required. Increasing wood harvests in regions experiencing higher demand may lead to unsustainable levels of resource extraction if forest resources are not carefully managed. This presents a challenge of balancing forest productivity with other factors, such as maintaining biodiversity and increasing carbon sequestration. Conversely, in scenarios such as SSP1‐RCP2.6, where demand growth is weak, a decrease in forest management intensity in many regions offers an opportunity to shift management towards practices which prioritize restoration and maintenance of ecosystem services. Given the uncertainty about the global socioeconomic trajectory, this highlights the need for forest management approaches which can respond adaptively to changes in global wood demand. Therefore, global coordination is necessary to align local forest management practices with broader environmental goals (MacDicken et al. 2015).

In this study, wood demand is predicted solely based on socioeconomic factors (population and GDP) and prices. The impact of policies such as those relating to wood‐based bioenergy is not included. Although woody biomass currently accounts for only 6% of the world's primary energy supply, this figure could increase as the world transitions away from fossil fuels (FAO 2016, 2024b). Woody biomass has the potential to meet 18% of the world's primary energy supply by 2050 (Lauri et al. 2014). The lack of this additional demand in our model means that the results may underestimate future pressures on forests, particularly in regions with a strong focus on wood‐based bioenergy for climate change mitigation. Additional wood demand could also come from a shift towards more wood‐based construction, which has the potential to store large amounts of carbon in buildings (Mishra et al. 2022; Zhao et al. 2022).

Policies such as the expansion of protected areas for biodiversity protection and implementation of GHG emission reduction measures through carbon pricing could also significantly impact future wood demand and forest management. In the scenarios presented here, protected areas were initialized from the World Database on Protected Areas (UNEP‐WCMC and IUCN 2025) and kept constant throughout the simulations. The expansion of protected areas could result in intensification of wood harvests outside of protected areas with negative consequences for biodiversity (Rosa et al. 2023). Ultimately, the impact of protected areas on forest management depends on their effectiveness, for example, to what degree wood harvests are permitted (Arneth et al. 2023). Other factors that could significantly impact global forest management, such as demand for carbon offsets through reforestation, forest restoration, and protection, are also not modeled here. Large‐scale afforestation and reforestation are likely to be necessary for cost‐effective climate change mitigation (Zhao et al. 2024), and may present trade‐offs with wood harvests not explored here (Daigneault et al. 2022).

While including additional sources of potential demand could significantly impact the projections presented here, these also come with additional uncertainties about which policies will likely be adopted in each scenario. For example, there is growing evidence that bioenergy from forest biomass is not as carbon neutral as is often assumed, which may limit its adoption (Peng et al. 2023; Schulze et al. 2012; Seo et al. 2024). However, higher demand for woody biomass could also incentivize afforestation and intensify forest management, resulting in a net increase in forest carbon stocks (Daigneault and Favero 2021; Favero et al. 2020). The scenarios presented here are based on historical patterns of wood demand and, therefore, require fewer assumptions about drivers of future wood demand. Nevertheless, exploring the impact of additional demand drivers would give a more complete view of how global forest management may evolve. Forthcoming work using LandSyMM should include further assumptions about land‐based climate change mitigation policies to address this limitation.

The projections presented here reflect potential forest yields under average climatic conditions and do not fully account for the increasing frequency and severity of extreme events such as droughts, heatwaves, and disturbances. These dynamics—along with emerging evidence of forest dieback and biome shifts (Boulton et al. 2022; Duffy et al. 2021)—represent critical uncertainties that may significantly constrain the capacity of forests to meet future wood demand. Our results suggest that under optimistic assumptions of CO_2_‐driven productivity gains and average climate trajectories, global forest management may be able to meet projected wood demand increases. However, these outcomes are subject to significant uncertainty, particularly with respect to extreme events, nutrient limitations, and potential biome shifts that are not fully captured in this modelling framework.

Currently, LandSyMM includes a representation of forest management based on rotational clear‐cutting. Modelling additional management practices such as thinning, reduced‐impact logging, and species selection could result in different outcomes than the ones presented here. There are inherent trade‐offs between different management practices in maximising forest ecosystem services such as wood harvesting, carbon sequestration, and biodiversity conservation (Brockerhoff et al. 2017; Felipe‐Lucia et al. 2018; Kolo et al. 2020). Understanding how management practices affect the balance of forest ecosystem services is critical for achieving global sustainability goals. Within the United Nations Sustainable Development Goals (SDGs), SDG 15 (Life on Land) emphasises protection and sustainable use of forests and calls for halting of deforestation and restoration of degraded forest ecosystems. Several other goals are fundamentally connected to forest management, including SDG 13 (Climate Action), which highlights the role of forests as carbon sinks and SDG 7 (Affordable and Clean Energy), which recognises the importance of wood‐based energy, particularly in low‐income countries. Achieving these goals in the context of growing demand for wood products will require carefully planned approaches to balance economic development and preservation of forest ecosystems.

As demand for wood products continues to increase through socioeconomic factors and policy drivers, intensifying forest management will present challenges to the long‐term health of forest ecosystems. This study highlights the importance of assessing the human impact on forests in the context of the whole land system rather than focusing solely on the forest sector. Competition between land uses for food, wood, and energy production can lead to complex and variable land use patterns. Global land use modelling frameworks such as LandSyMM are essential for exploring potential future pathways for sustainable management of the land system. Meeting the growing demand for food, materials, and energy without putting additional strain on natural ecosystems requires careful consideration of the benefits and trade‐offs of different land management practices.

## Author Contributions

**Bartlomiej Arendarczyk:** conceptualization, data curation, formal analysis, investigation, methodology, software, visualization, writing – original draft, writing – review and editing. **Sam Rabin:** conceptualization, investigation, methodology, software, writing – review and editing. **Daniel Bampoh:** investigation, software, writing – review and editing. **Almut Arneth:** conceptualization, funding acquisition, methodology, supervision, writing – review and editing. **Mark Rounsevell:** conceptualization, funding acquisition, methodology, supervision, writing – review and editing. **Peter Alexander:** conceptualization, funding acquisition, methodology, software, supervision, writing – review and editing.

## Conflicts of Interest

The authors declare no conflicts of interest.

## Supporting information


**Data S1:** gcb70573‐sup‐0001‐Supinfo.pdf.

## Data Availability

Model output data can be found at https://doi.org/10.5281/zenodo.14004333. Model input data can be obtained from public sources including the FAO (https://www.fao.org/faostat), World Bank (https://data.worldbank.org), and IIASA (https://tntcat.iiasa.ac.at/SspDb). Data from LPJ‐GUESS can be provided upon request. Model source code is available at https://git.ecdf.ed.ac.uk/lul/plumv2/‐/tree/ForestryPaper.
