# Supplementary material for: Response of Global Forest Management to Changes in Wood Demand
Source: Glob Chang Biol. 2025 Nov 4;31(11):e70573. doi: 10.1111/gcb.70573 (PMC12584040; doi:10.1111/gcb.70573)
Supplement: Supplementary file 1 — Data S1: gcb70573‐sup‐0001‐Supinfo.pdf. [file GCB-31-e70573-s001.pdf]

# Supplementary Information for “Response of global forest management to changes in wood demand”

## Supplementary Methods

### Data cleaning

We applied data cleaning steps to correct inconsistencies in the wood production and trade data from FAOSTAT. Firstly, we assumed that trade figures are more accurate than reported production (Kallio & Solberg, 2018). Assuming no re-exporting of imported goods, exports impose a lower limit on domestic production. Therefore, production was set to the export amount in cases where exports exceeded reported production. Secondly, under-reporting of wood harvests is a known issue in the FAO data, particularly in developing countries (Kallio & Solberg, 2018). We corrected the reported harvest of industrial roundwood by estimating how much industrial roundwood was harvested in each country based on the reported production of derived items:

$$harvest_{est} = \sum_i production_i - \sum_j imports_j \qquad (1)$$

where $\mathrm{harvest_{est}}$ is the estimated industrial roundwood harvest. Here, $i$ includes sawnwood, wood-based panels, veneer sheets, wood chips, particles and residues, wood pulp, and wood pellets and other agglomerates. Additionally, $j$ includes industrial roundwood and wood chips, particles and residues. Items in $j$ represent potential raw materials for production of items in $i$ and are therefore subtracted from total production to avoid including production from imported materials. To correct the under-reporting of industrial roundwood harvests, we assumed that domestic harvest is equal to $\max(\mathrm{harvest_{fao}}, \mathrm{harvest_{est}})$ where $\mathrm{harvest_{fao}}$ is the reported harvest in the FAO data. Wood fuel harvest was taken as reported, and no corrections were applied. While this may lead to underestimation of wood fuel harvests, no additional data was available to correct for this.

In some countries, simulated wood yields were insufficient to match reported wood production. To avoid issues during PLUM spin-up, we adjusted initial wood trade and

production levels. We calculated the potential maximum wood production in each country using the yield tables, assuming a rotation period of 10 years or more. For each grid cell, the highest yielding rotation period was selected, and the yield was multiplied by the reported forest area in that grid cell, including both managed and unmanaged forest. Potential production was aggregated to the country level and compared against reported production. Where reported production exceeded the maximum potential production, it was adjusted down, and the difference was shifted to imports. This procedure does not affect countries' wood demand but ensures that initialised production can be met to avoid solution failure in the land use optimiser.

## Wood demand model

The following MAIDADS equations are adapted from Gouel & Guimbard (2019) to simulate wood demand:

$$x_i = \frac{\delta_i + \tau_i e^{\omega u}}{1+e^{\omega u}} + \frac{1}{p_i}\frac{\alpha_i + \beta_i e^{u}}{1+e^{u}}\left(m - \sum_j p_j \frac{\delta_i + \tau_i e^{\omega u}}{1+e^{\omega u}}\right) \qquad (2.1)$$

$$\sum_i \frac{\alpha_i + \beta_i e^{u}}{1+e^{u}} ln\left(x_i - \frac{\delta_i + \tau_i e^{\omega u}}{1+e^{\omega u}}\right) - u = \kappa \qquad (2.2)$$

where $x_i$ is the demand of item $i \in$ {industrial roundwood, wood fuel} and $u$ is the utility. Parameters $\alpha, \beta, \delta, \tau, \omega$, and $\kappa$ are fitted using a similar procedure to the one described in Gouel & Guimbard (2019) (Supplementary Table 1). To reduce the impact of outliers on the model fit, we removed countries where industrial roundwood or wood fuel consumption was greater than 4 $m^3$ $capita^{-1}$ $year^{-1}$. We also removed countries with a population of less than one million as these countries include small island nations with unusual consumption patterns and less reliable data. A total of 147 countries was included in the model fitting. During a model run, demand predictions were rebased using a constant offset to match reported demand in 2020.

| Commodity | α | β | δ | τ |
|---|---|---|---|---|
| Industrial roundwood | 0.6594210 | 0 | 0.0245140 | 0 |
| Wood Fuel | 0.0351533 | 0 | 0.4862020 | 0.0725917 |
| Residual | 0.3054260 | 1 | 0 | 2.4658700 |
| Other parameters: κ = -3.3648, ω = 1.47775 | | | | |

Supplementary Table 1 – MAIDADS parameters for the fitted wood demand model.

## Crop yield calibration

Crop yields were simulated by LPJ-GUESS version 4.1 as described in Alexander et al. (2018) but using bias-adjusted ISIMIP-3b, CMIP6-based atmospheric climate forcings for the MRI-ESM2-0 model (Lange & Büchner, 2021; Yukimoto et al., 2019). Yields were simulated using a factorial experiment involving three nitrogen fertiliser application levels (0 kg $ha^{-1}$, 200 kg $ha^{-1}$, and 1000 kg $ha^{-1}$) and two irrigation levels (no irrigation and wilting-point). Simulated yields were calibrated on a country level to observed yields from FAOSTAT (FAO, 2024) for 2005-2014, using historical nitrogen fertiliser use, irrigation, and crop distributions. Calibration was required, firstly to convert simulated yields from dry-matter units to wet-matter units (i.e. as reported in FAOSTAT), and secondly to account for systematic biases in simulated yields.

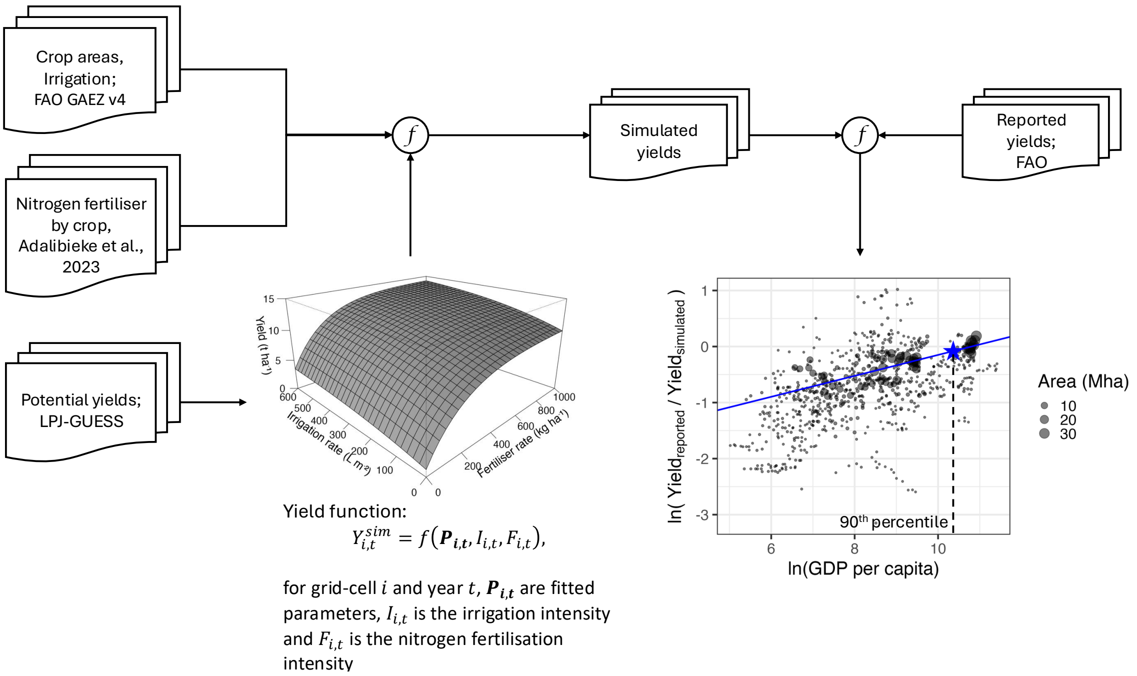


Supplementary Figure 1 - Diagram showing the crop yield calibration procedure. Yield response functions were fitted using potential yields from a factorial experiment in LPJ-

GUESS. Expected yields were calculated using reported crop areas, nitrogen fertilisation and irrigation between 2005 and 2014. Calibration factors were calculated from country-level aggregated expected yields and reported yields. Global calibration factors were estimated using OLS regression.

Observed gridded crop areas were taken from GAEZ v4 (FAO, 2022) which includes the distribution of 27 crop groups in 2010, divided into irrigated and rainfed areas. Annual gridded nitrogen fertiliser application were obtained from Adalibieke et al. (2023). Reported country-level crop yields were taken from FAOSTAT (FAO, 2024). A global yield calibration factor was calculated for 143 crops. For each crop and year, yields were simulated using a yield response function fitted using the yield tables from LPJ-GUESS (Equation 3). For each crop:

$$Y_{i,t}^{sim} = f\left(\boldsymbol{P_{i,t}}, I_{i,t}, F_{i,t}\right), \quad (3)$$

where $Y_{i,t}^{sim}$ is the simulated yield at grid-cell $i$ in year $t$, $\boldsymbol{P_{i,t}}$ are fitted parameters, $I_{i,t}$ is the irrigation intensity and $F_{i,t}$ is the nitrogen fertilisation intensity. Irrigation was assumed to be 0 for rainfed areas and 1 (i.e. fully irrigated) for irrigated areas. Function $f$ defines an exponential yield surface as detailed in Alexander et al. (2018) (Supplementary Figure 1). The same function is used to simulate crop yield responses during a model run in PLUM.

Simulated yields were calculated for each year between 2005 and 2014. Crop areas and irrigation were assumed to remain constant as per the GAEZ 2010 dataset. Nitrogen fertilisation was updated for each year as per Adalibieke et al. (2023). Simulated yields were aggregated to the country level and a calibration factor was calculated for each crop and country as a ratio between reported and simulated yields (Equation 4). For each crop:

$$k_{c,t} = \frac{Y_{c,t}^{obs}}{Y_{c,t}^{sim}}, \quad (4)$$

where $Y_{c,t}^{sim}$ is the simulated mean yield and $Y_{c,t}^{obs}$ is the reported mean yield for country $c$ in year $t$, and $k_{c,t}$ is the calibration factor.

We assumed that differences in calibration factors between countries for each crop are due to random error. Therefore, a single, global yield calibration factor was calculated for each crop using OLS regression, weighted by crop area in each country (Equation 5). While we accounted for nitrogen fertiliser input and irrigation, other factors which could affect yields such as pesticide use and mechanisation were not simulated in LPJ-GUESS and therefore not included in Equation 3. We found that, for most crops, yield calibration factors were positively correlated with GDP per capita, suggesting higher agricultural inputs in high income countries. Lower agricultural intensities in low income countries result in a yield gap that introduces a bias in the calculation of the yield calibration factors. Therefore, we included GDP per capita in the regression to control for agricultural inputs other than fertiliser and irrigation. For each crop:

$$\ln(k_{c,t}) = \alpha + \beta \ln(g_{c,t}) + \epsilon_{c,t}, \qquad (5)$$

where $g_{c,t}$ is the GDP per capita for country $c$ in year $t$, α and β are parameters, and $\epsilon_{c,t}$ is the error term. Calibration factors and GDP per capita were log-transformed to ensure that the derived global calibration factor is positive. Reported yields more than 3 standard deviations from the global mean were excluded.

To remove the bias of lower agricultural inputs in low-income countries, global calibration factors were calculated using the sample 90$^{th}$ percentile GDP per capita (Equation 6). For each crop:

$$k^{glo} = exp(\alpha + \beta \ln(g^{*})), \qquad (6)$$

where $k^{glo}$ is the global calibration factor and $g^{*}$ is the sample 90$^{th}$ percentile GDP per capita.

If β was not significant at the alpha=0.05 level or was negative (implying decreasing yields with income), the global calibration factor was calculated using a simplified model which excludes GDP per capita (Equations 7and 8). For each crop:

$$\ln(k_{c,t}) = \alpha + \epsilon_{c,t}; \qquad (7)$$

$$k^{glo} = exp(\alpha). \qquad (8)$$

Finally, an area-weighted average calibration factor was calculated for each CFT and country. Supplementary Table 2 shows an example calculation for C3 cereals in the United Kingdom.

| Crop | Area | Calibration Factor |
|---|---|---|
| Wheat | 1.884 | 0.791 |
| Barley | 1.008 | 0.827 |
| Oats | 0.127 | 0.784 |
| Triticale | 0.014 | 0.995 |
| Rye | 0.006 | 0.703 |
| Grain mixed | 0.005 | 0.975 |
| C3 Cereals area-weighted average | | 0.804 |

Supplementary Table 2 – Calculation of the yield calibration factor for C3 cereals for the United Kingdom.

## Dietary preferences

To model changes in dietary preferences, we adjusted the $\alpha$ and $\tau$ parameters in MAIDADS (Gouel & Guimbard, 2019) which control the relationship between income and the subsistence and discretionary demand of each food commodity. Both parameters were interpolated linearly between 2020 and 2050 between default values (fitted on historical data) and target values. Target parameters values were calculated using adjustment ratios $r_i$ (Supplementary Table 3):

$$\alpha_i^* = r_i \cdot k \cdot \alpha + (1 - k) \cdot \alpha; \qquad (9.1)$$

$$\tau_i^* = r_i \cdot k \cdot \tau + (1 - k) \cdot \tau, \qquad (9.2)$$

where $\alpha_i^*$ and $\tau_i^*$ are the target parameters for food group $i$, $\alpha$ and $\tau$ are the reference parameters, and $k$ is a scenario specific value (from 0 to 1) that determines the degree of

change in dietary preferences. The following values of $k$ were used in this study: 1 for SSP1, 0 for SSP3, 0.5 for SSP4, and 0 for SSP5. Given the constraint that $\sum_i \alpha_i = 1$ where $i$ is the commodity group, we assumed that the $\alpha_{nonfood} = 1 - \sum_{i \neq nonfood} \alpha_i$.

| Commodity Group | EAT Lancet (kcal/day) | Reference (kcal/day) | $r_i$ |
|---|---|---|---|
| Cereals & Starchy Roots | 995 | 1163 | 0.86 |
| Fruit & Vegetables | 413 | 398 | 1.04 |
| Monogastrics | 112 | 348 | 0.32 |
| Oil crops | 782 | 433 | 1.81 |
| Pulses | 201 | 29 | 7.01 |
| Ruminants | 239 | 461 | 0.52 |
| Sugar | 140 | 395 | 0.36 |

Supplementary Table 3 – EAT Lancet (Willett et al., 2019) target diet, reference diet, and adjustment ratios ($r_i$).

# Model parameterisation

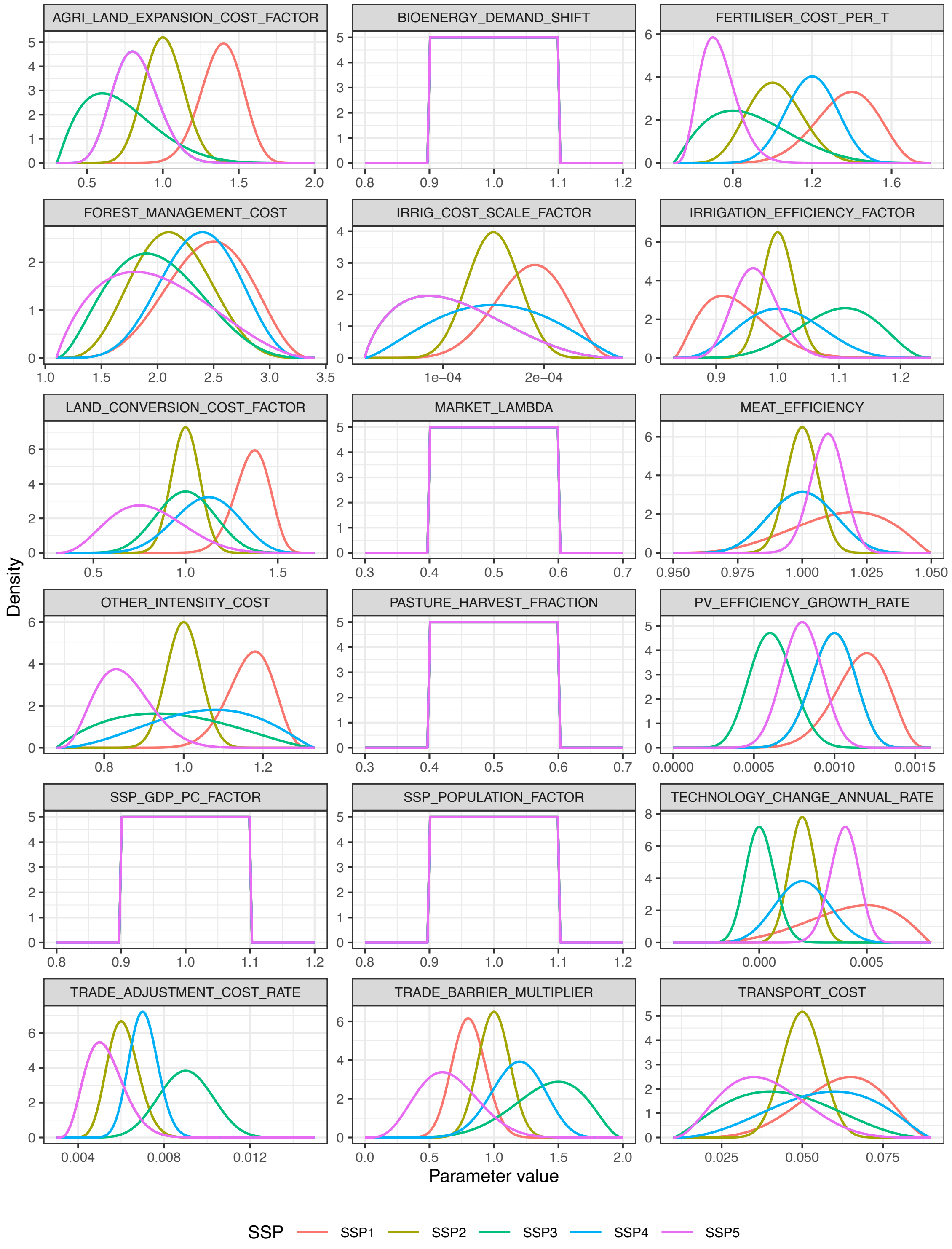


Supplementary Figure 2 – Distribution of sampled input parameters.

# Supplementary Results

## Price elasticity of wood demand

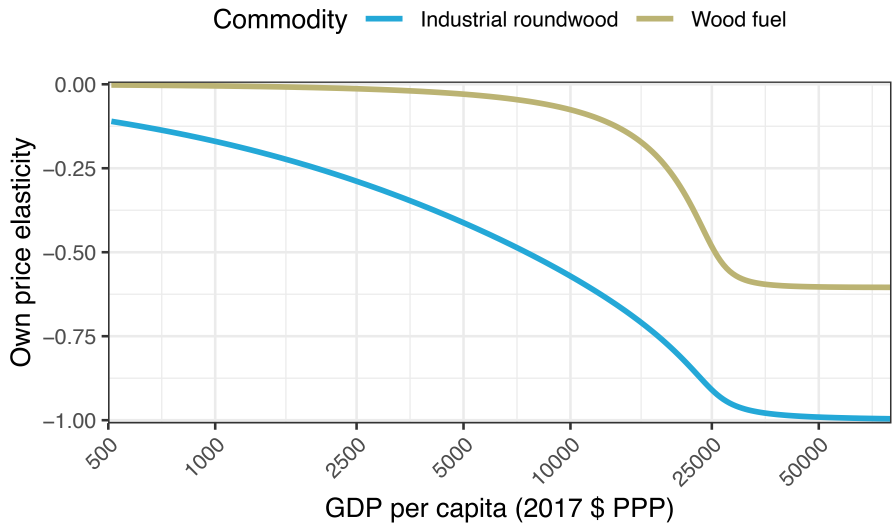


Supplementary Figure 3 - Own price elasticity of wood demand for industrial roundwood (blue line) and wood fuel (brown line) as a function of GDP per capita.

## Model validation

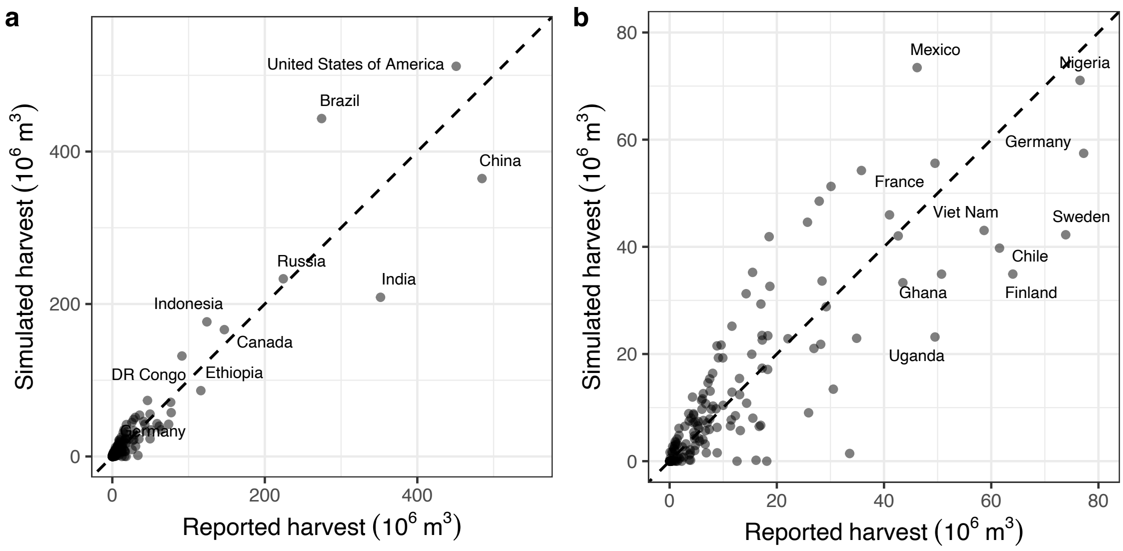


Supplementary Figure 4 - Reported simulated total wood harvest by country in 2020 for (a) all countries and (b) countries with reported harvest below 80 million $m^3$. Dashed lines show y=x. Reported harvests were obtained from FAOSTAT (FAO, 2024).

## Wood demand

| | | Global demand; $10^6$ $m^3$ (90% CI) | | |
|---|---|---|---|---|
| Scenario | Year | Industrial roundwood | Wood fuel | Total wood |
| Baseline | 2020 | 2176 | 1959 | 4135 |
| | | | | |
| SSP1-RCP2.6 | 2060 | 4079 (3738 - 4496) | 1396 (1262 - 1521) | 5502 (5007 - 6011) |
| SSP2-RCP4.5 | | 4189 (3736 - 4582) | 1931 (1740 - 2153) | 6120 (5496 - 6682) |
| SSP3-RCP7.0 | | 3592 (3075 - 4027) | 2712 (2427 - 3027) | 6327 (5563 - 6983) |
| SSP4-RCP6.0 | | 3359 (3032 - 3746) | 2383 (2161 - 2614) | 5799 (5238 - 6314) |
| SSP5-RCP8.5 | | 5058 (4262 - 5988) | 1556 (1313 - 1792) | 6603 (5575 - 7780) |
| | | | | |
| SSP1-RCP2.6 | 2100 | 4019 (3655 - 4397) | 1236 (1123 - 1335) | 5257 (4778 - 5732) |
| SSP2-RCP4.5 | | 5237 (4632 - 5745) | 1826 (1644 - 1980) | 7070 (6276 - 7725) |
| SSP3-RCP7.0 | | 5105 (4438 - 5773) | 3193 (2838 - 3609) | 8337 (7351 - 9296) |
| SSP4-RCP6.0 | | 3592 (3223 - 4045) | 2830 (2565 - 3104) | 6478 (5853 - 7103) |
| SSP5-RCP8.5 | | 6101 (4992 - 7339) | 1757 (1447 - 2096) | 7862 (6439 - 9434) |

Supplementary Table 4 – Baseline and simulated future global wood demand. Median values and 90% CIs are shown for 30 ensemble members per scenario.

## Forest area

| Scenario | Year | Timber forest area (Mha) | Unmanaged forest area (Mha) |
|---|---|---|---|
| Baseline | 2020 | 1460 | 2642 |
| | | | |
| SSP1-RCP2.6 | 2060 | 1458 (1451 - 1472) | 2641 (2624 - 2648) |
| SSP2-RCP4.5 | | 1485 (1468 - 1507) | 2556 (2476 - 2593) |
| SSP3-RCP7.0 | | 1499 (1470 - 1518) | 2493 (2367 - 2565) |
| SSP4-RCP6.0 | | 1481 (1457 - 1514) | 2582 (2512 - 2623) |
| SSP5-RCP8.5 | | 1511 (1473 - 1581) | 2444 (2353 - 2537) |
| | | | |
| SSP1-RCP2.6 | 2100 | 1467 (1455 - 1488) | 2628 (2602 - 2644) |
| SSP2-RCP4.5 | | 1523 (1496 - 1562) | 2459 (2380 - 2518) |
| SSP3-RCP7.0 | | 1550 (1519 - 1598) | 2303 (2092 - 2429) |
| SSP4-RCP6.0 | | 1501 (1462 - 1538) | 2554 (2474 - 2608) |
| SSP5-RCP8.5 | | 1533 (1478 - 1645) | 2423 (2289 - 2511) |

Supplementary Table 5 – Baseline and simulated future timber forest and unmanaged forest global area. Median values and 90% CIs are shown for 30 ensemble members per scenario.

# Forest management and harvest

| Scenario | Year | Management intensity | Wood harvest ($10^6$ $m^3$) | Yield ($m^3$ $ha^{-1}$) |
|---|---|---|---|---|
| Baseline | 2020 | 0.0180 | 4103 | 2.81 |
| | | | | |
| SSP1-RCP2.6 | 2060 | 0.0225 (0.0192 - 0.0272) | 5459 (4979 - 5934) | 3.73 (3.44 - 4.04) |
| SSP2-RCP4.5 | | 0.0248 (0.0209 - 0.0298) | 6087 (5424 - 6596) | 4.07 (3.71 - 4.40) |
| SSP3-RCP7.0 | | 0.0258 (0.0212 - 0.0314) | 6249 (5509 - 6884) | 4.16 (3.75 - 4.54) |
| SSP4-RCP6.0 | | 0.0235 (0.0199 - 0.0272) | 5823 (5242 - 6343) | 3.93 (3.61 - 4.22) |
| SSP5-RCP8.5 | | 0.0270 (0.0210 - 0.0351) | 6695 (5637 - 7848) | 4.40 (3.87 - 4.94) |
| | | | | |
| SSP1-RCP2.6 | 2100 | 0.0195 (0.0167 - 0.0233) | 5265 (4777 - 5738) | 3.58 (3.30 - 3.87) |
| SSP2-RCP4.5 | | 0.0245 (0.0206 - 0.0293) | 7075 (6273 - 7721) | 4.59 (4.20 - 4.94) |
| SSP3-RCP7.0 | | 0.0297 (0.0241 - 0.0367) | 8267 (7301 - 9169) | 5.27 (4.82 - 5.72) |
| SSP4-RCP6.0 | | 0.0215 (0.0184 - 0.0247) | 6565 (5895 - 7173) | 4.39 (4.02 - 4.67) |
| SSP5-RCP8.5 | | 0.0236 (0.0185 - 0.0306) | 8118 (6636 - 9749) | 5.22 (4.57 - 5.88) |

Supplementary Table 6 – Baseline and simulated mean forest management intensity, total wood harvest, and mean yield. Median values and 90% CIs are shown for 30 ensemble members per scenario.

## Changes in timber forest area

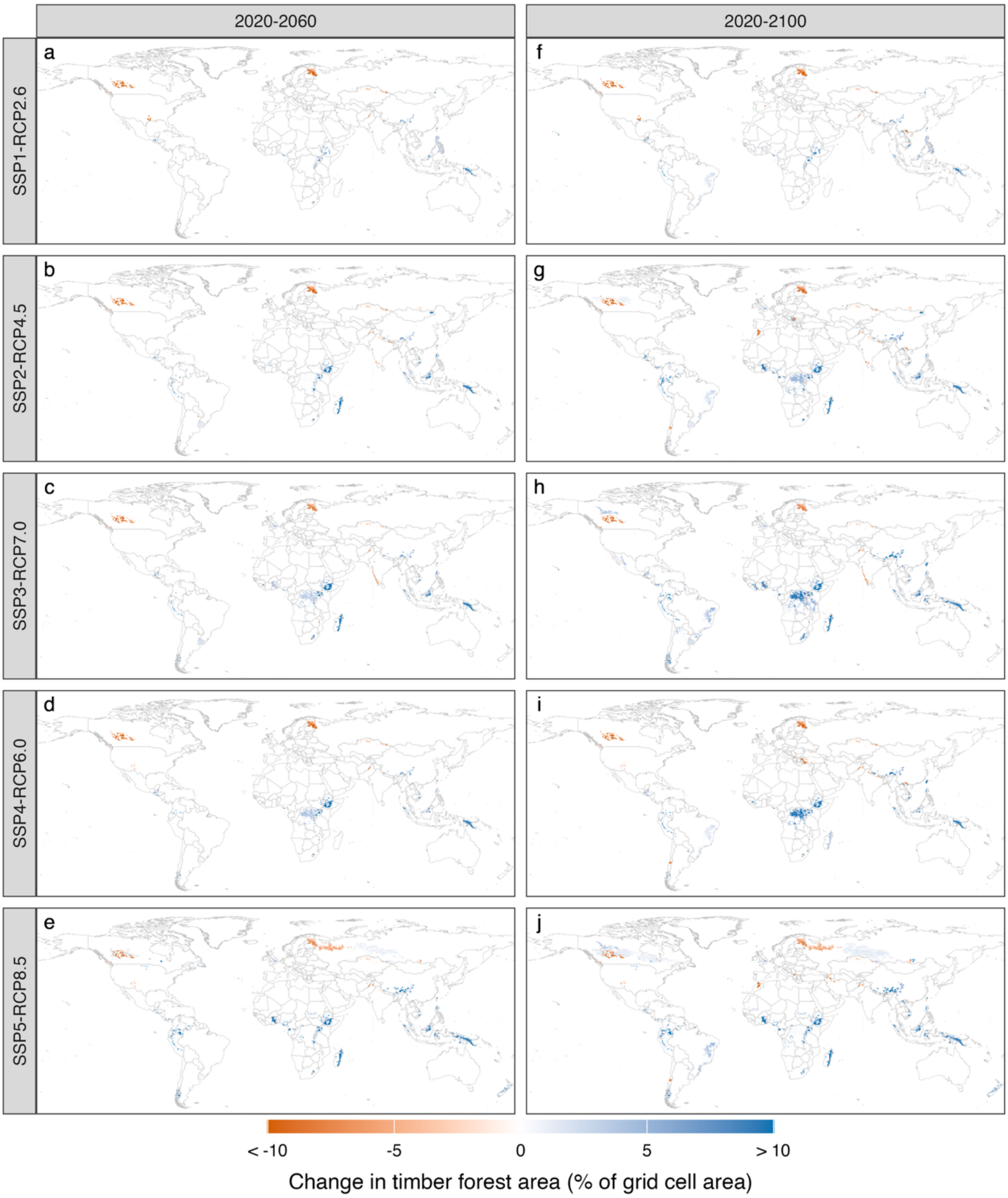


Supplementary Figure 5 - Change in timber forest area for 2020-2060 (a, b, c, d, e) and 2020-2100 (f, g, h, i, j). Changes are calculated as the median difference in area across all ensemble members (*n*=30). Map lines delineate study areas and do not necessarily depict accepted national boundaries.

## Changes in forest management intensity

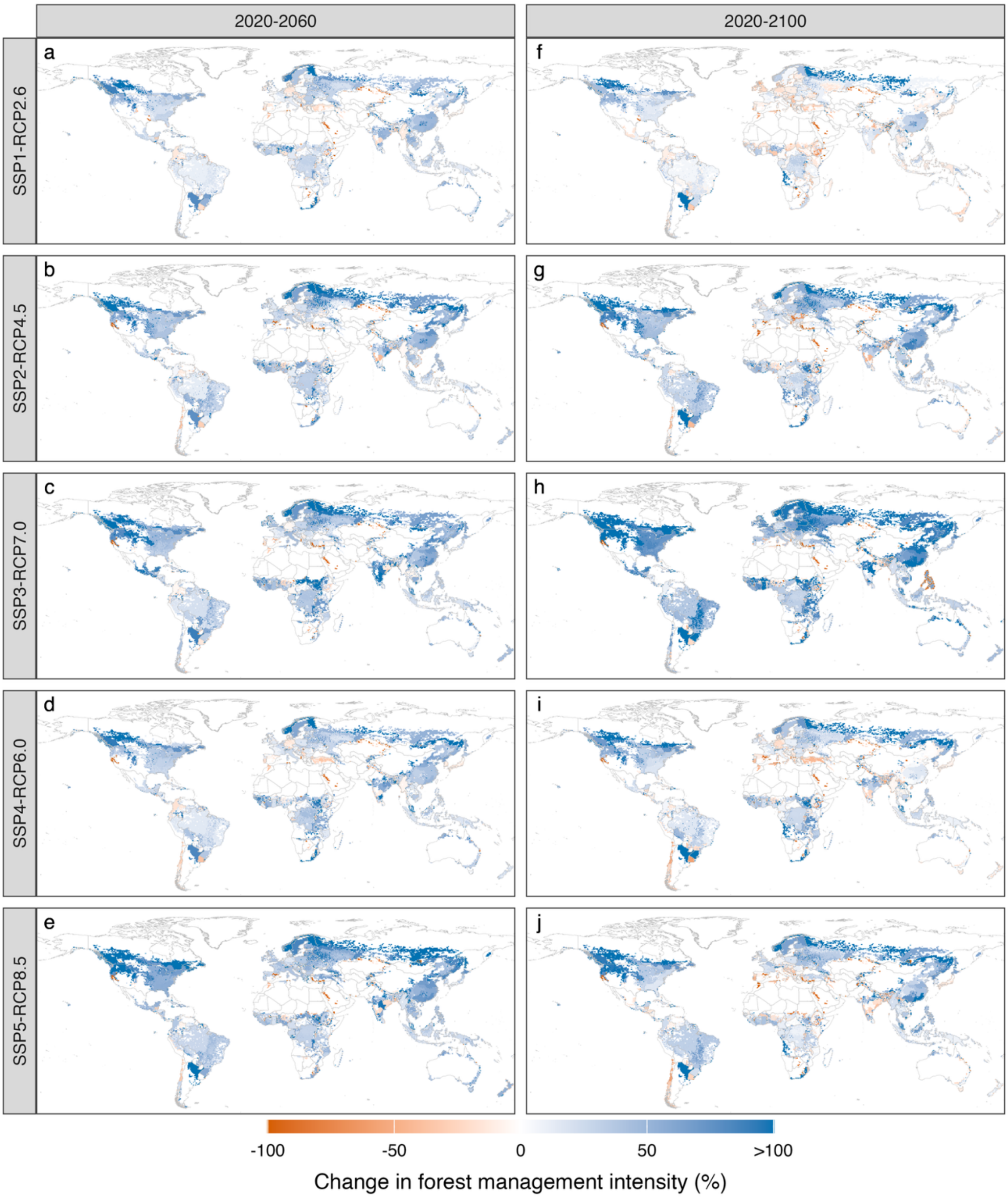


Supplementary Figure 6 - Change in forest management intensity for 2020-2060 (a, b, c, d, e) and 2020-2100 (f, g, h, i, j). Changes are calculated as the median percentage difference from the baseline value in 2020 across all ensemble members (n=30). Map lines delineate study areas and do not necessarily depict accepted national boundaries.

## Changes in wood harvests

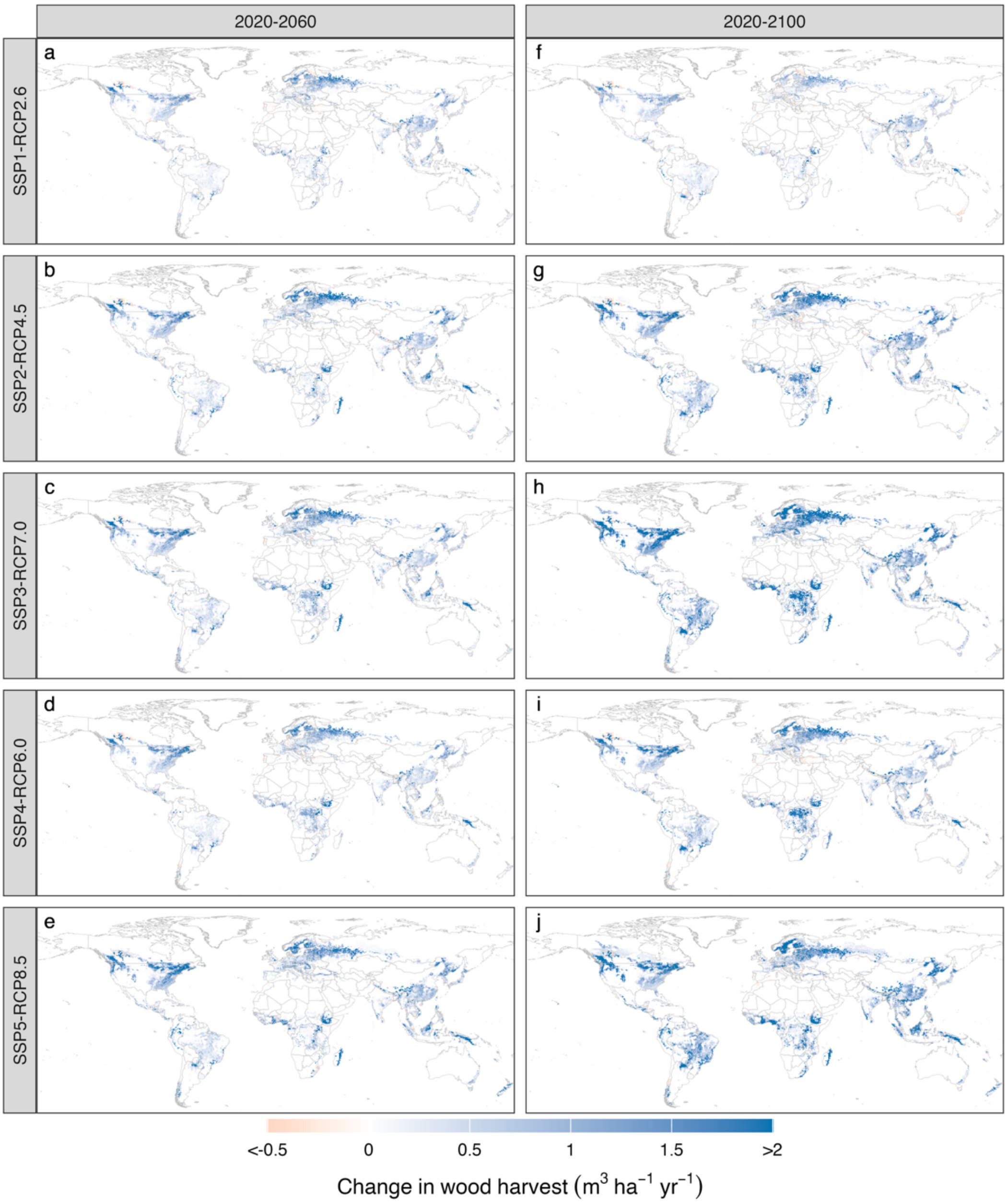


Supplementary Figure 7 - Change in wood harvests for 2020-2060 (a, b, c, d, e) and 2020-2100 (f, g, h, i, j). Changes are calculated as the median difference in harvests across all ensemble members (*n*=30). Map lines delineate study areas and do not necessarily depict accepted national boundaries.

# References

Adalibieke, W., Cui, X., Cai, H., You, L., & Zhou, F. (2023). Global crop-specific nitrogen fertilization dataset in 1961–2020. *Scientific Data*, *10*(1), 617. https://doi.org/10.1038/s41597-023-02526-z

Alexander, P., Rabin, S., Anthoni, P., Henry, R., Pugh, T. A. M., Rounsevell, M. D. A., & Arneth, A. (2018). Adaptation of global land use and management intensity to changes in climate and atmospheric carbon dioxide. *Global Change Biology*, *24*(7), 2791–2809. https://doi.org/10.1111/gcb.14110

FAO. (2022). *GAEZ v4 Data Portal* [Dataset]. https://gaez.fao.org/

FAO. (2024). *FAOSTAT*. https://www.fao.org/faostat/en/

Gouel, C., & Guimbard, H. (2019). Nutrition Transition and the Structure of Global Food Demand. *American Journal of Agricultural Economics*, *101*(2), 383–403. https://doi.org/10.1093/ajae/aay030

Kallio, A. M. I., & Solberg, B. (2018). On the Reliability of International Forest Sector Statistics: Problems and Needs for Improvements. *Forests*, *9*(7), 407. https://doi.org/10.3390/f9070407

Lange, S., & Büchner, M. (2021). *ISIMIP3b bias-adjusted atmospheric climate input data* (Version 1.1) [Dataset]. ISIMIP Repository. https://doi.org/10.48364/ISIMIP.842396.1

Willett, W., Rockström, J., Loken, B., Springmann, M., Lang, T., Vermeulen, S., Garnett, T., Tilman, D., DeClerck, F., Wood, A., Jonell, M., Clark, M., Gordon, L. J., Fanzo, J., Hawkes, C., Zurayk, R., Rivera, J. A., De Vries, W., Majele Sibanda, L., … Murray, C. J. L. (2019). Food in the Anthropocene: The EAT–Lancet Commission on healthy diets from sustainable food systems. *The Lancet*, *393*(10170), 447–492. https://doi.org/10.1016/S0140-6736(18)31788-4

Yukimoto, S., Kawai, H., Koshiro, T., Oshima, N., Yoshida, K., Urakawa, S., Tsujino, H., Deushi, M., Tanaka, T., Hosaka, M., Yabu, S., Yoshimura, H., Shindo, E., Mizuta, R., Obata, A., Adachi, Y., & Ishii, M. (2019). The Meteorological Research Institute Earth System Model Version 2.0, MRI-ESM2.0: Description and Basic Evaluation of the Physical Component. *Journal of the Meteorological Society of Japan. Ser. II*, *advpub*, 2019–2051. https://doi.org/10.2151/jmsj.2019-051
